# Multi-trait selection in multi-environments for performance and stability in cassava genotypes

**DOI:** 10.3389/fpls.2023.1282221

**Published:** 2023-10-30

**Authors:** Juraci Souza Sampaio Filho, Tiago Olivoto, Marcos de Souza Campos, Eder Jorge de Oliveira

**Affiliations:** ^1^ Federal University of the Recôncavo da Bahia, Cruz das Almas, Bahia, Brazil; ^2^ Department of Crop Science, Federal University of Santa Catarina, Florianópolis, Brazil; ^3^ Embrapa Mandioca e Fruticultura, Cruz das Almas, Bahia, Brazil

**Keywords:** *Manihot esculenta* Crantz, genotype vs. environment interaction, mixed model, breeding, stability

## Abstract

Genotype-environment interaction (GEI) presents challenges when aiming to select optimal cassava genotypes, often due to biased genetic estimates. Various strategies have been proposed to address the need for simultaneous improvements in multiple traits, while accounting for performance and yield stability. Among these methods are mean performance and stability (MPS) and the multi-trait mean performance and stability index (MTMPS), both utilizing linear mixed models. This study’s objective was to assess genetic variation and GEI effects on fresh root yield (FRY), along with three primary and three secondary traits. A comprehensive evaluation of 22 genotypes was conducted using a randomized complete block design with three replicates across 47 distinct environments (year x location) in Brazil. The broad-sense heritability ( $H^{2}$ ) averaged 0.37 for primary traits and 0.44 for secondary traits, with plot-based heritability ( $h_{mɡ}^{2})$ consistently exceeding 0.90 for all traits. The high extent of GEI variance ( $\sigma_{ɡxe}^{2}$ ) demonstrates the GEI effect on the expression of these traits. The dominant analytic factor $(FA_{3}$ ) accounted for over 85% of the total variance, and the communality (ɧ) surpassed 87% for all traits. These values collectively suggest a substantial capacity for genetic variance explanation. In Cluster 1, composed of remarkably productive and stable genotypes for primary traits, genotypes BRS Novo Horizonte and BR11-34-69 emerged as prime candidates for FRY enhancement, while BRS Novo Horizonte and BR12-107-002 were indicated for optimizing dry matter content. Moreover, MTMPS, employing a selection intensity of 30%, identified seven genotypes distinguished by heightened stability. This selection encompassed innovative genotypes chosen based on regression variance index ( $S_{di}^{2}$ , $R^{2}$ , and RMSE) considerations for multiple traits. In essence, incorporating methodologies that account for stability and productive performance can significantly bolster the credibility of recommendations for novel cassava cultivars.

## 1 Introduction

Cassava (*Manihot esculenta* Crantz), a member of the Euphorbiaceae family, displays high genetic variability. It is one of the primary agricultural species and holds significant industrial potential, particularly in the starch extraction from its roots, which is considered the species’ primary product. However, all parts of the plant can be commercially utilized or explored as genetic plant resources (Ceballos et al., 2012).

With a global production of 302.6 million tons cultivated across 28.2 million hectares and an average yield of 10.7 t ha^-1^, cassava is grown in over 90 countries. It ranks as the fourth largest caloric source and the second most important source of starch globally, playing a crucial role in ensuring food security (Stapleton, 2012; FAO, 2020; Ceballos et al., 2021). Given its pervasive cultivation across continents, and in the face of global climate change, coupled with various environmental factors, existem significant influences and associations affect cassava’s yield, profitability and stability. Additionally, genetic factors such as asynchronous flowering, heterozygosity, inbreeding, and low seed set pose potential challenges to cassava improvement progress (Ceballos et al., 2020). Hence, overcoming these challenges and attaining successful production hinges on effectively employing modern breeding strategies across diverse and specific agronomically relevant environments.

In breeding programs, the selection process involves conducting numerous trials in the target production centers to recommend new varieties across multiple crop years. In general, it takes 8 to 10 years to develop a new cassava cultivar, considering the following stages in the breeding program: i) crossing to obtain progenies; ii) seedling production and field transplantation; iii) clonal evaluation trials (CET), the initial stage of agronomic evaluation characterized by a few environments and a high number of clones; iv) preliminary yield trials (PYT), represented by 2-3 field replications, fewer plants per plot (1 row with 6-8 plants), and 1-2 locations; v) advanced yield trials (AYT), with 3 replications, larger plots (4 rows with 16-24 plants), and 3-5 locations; vi) uniform yield trials (UYT), featuring 3-4 field replications, larger plots (4-6 rows with 40-60 plants), and 8-12 evaluation locations; and vii) cultivar value and use trials (VCU). Therefore, generally, starting from the AYT trials, cassava clones are already evaluated in multi-environment tests (METs) over several years to assess genotype-environment interaction (GEI) (Oliveira et al., 2012; Wolfe et al., 2016; Ceballos et al., 2020). These trials aim to enhance our understanding of variables, influencing phenotypic expression and identify genotypes with superior performance and stability in order to mitigate the risk of crop failure and low adoption rates for new varieties.

METs are conducted annually to examine the genotype × environment interaction (GEI), a natural phenomenon that incorporates the effects of genotypes and environments as well as the non-consistency in genotype ranking for specific traits. Assessing the importance of genetic and environmental factors is particularly important in the selection process, especially for traits with low heritability, as they may potentially exhibit more pronounced GEI effects (Malosetti et al., 2013; Olivoto et al., 2019a).

In recent decades, a range of statistical methods have been developed to model GEI, identify genotypes that are stable and well-adapted, and select target environments, including mega-environments that share similar climatic patterns. These methods vary from simple nonparametric analysis-of-variance techniques with fixed and/or linear-bilinear effects to more robust univariate and multivariate parametric methods. These advancements aim to achieve more advanced results and a more systematic characterization of GEI (Yates and Cochran, 1938; Finlay and Wilkinson, 1963; Eberhart and Russell, 1966; Gauch and Zobel, 1988; Yan et al., 2000). More recently, linear mixed-effects models (LMM) have gained popularity, allowed estimation of best linear unbiased predictions (BLUPs), and provided reliable estimates of variance components and genetic values. LMMs also enable modeling heterogeneity of genetic variances and correlations between environments with contrasting conditions and unbalanced data. This capability generates robust predictions of genotypic values and reduces noise, which is especially valuable given the nature of MET (Smith et al., 2005; Hu, 2015; Van Eeuwijk et al., 2016).

In the final phases of breeding programs, a significant challenge emerges due to the inherent imbalance in trials caused by replacing underperforming clones with new ones for testing in subsequent years. This results in varying numbers of genotypes being evaluated annually and across different locations, leading to a pronounced imbalance in the phenotypic data. Conventional approaches to studying the GEI face difficulties when dealing with such imbalanced data, resulting in disparate variances and covariances of fitted mean phenotypic values. These variances and covariances play a crucial role for breeders in selecting genotypes for further assessment or recommending them as new varieties. Furthermore, innovative mixed model-based approaches, like analytical factor analysis, facilitate the integration of kinship information by employing covariance matrices of random genetic effects (Bernardo, 2020).

Beyond exhibiting high agronomic potential in specific target environments, novel cassava varieties must also demonstrate phenotypic stability to gain recommendation and adaption across wider geographical regions, whether for industrial use or direct consumption. Nevertheless, selecting genotypes with strong performance across multiple traits simultaneously presents a complex challenge due to undesirable correlations stemming from intricate trait relationships and genetic architectures (Egesi et al., 2007; Van Eeuwijk et al., 2016; Olivoto et al., 2019a). Most practical breeding efforts have historically focused on approaches that solely focus on individual traits in isolation, like truncated selection, can prove inadequate when populations lack the necessary variation for all traits of interest, necessitate larger population sizes, or encounter unfavorable correlations between pivotal traits. Given the need for simultaneous genetic gains in multiple traits, with rapid progress and enhanced reliability in recommendations, various methods have been proposed, including: FAI BLUP (a multi-trait index based on factor analysis and genotype-ideotype distance – Rocha et al., 2018), MTSI (a multi-trait stability index – Olivoto et al., 2019a), GYT (genotype by yield × traits – Yan and Frégeau-Reid, 2018), and MGIDI (a multi-trait genotype-ideotype distance index - Olivoto and Nardino, 2021). However, there are limited reports that associate the study of GEI, performance, adaptability, and genotypic stability for multi-trait evaluations with unbalanced data in cassava trials.

To ensure the delivery of varieties that meet consumer expectations and gain high adoption in the agricultural sector, the selection process should consider not only yield-related attributes but also characteristics associated with plant architecture, reproductive capacity, soil coverage to minimize weed interference, and root quality for improved commercial acceptance, particularly when the roots are intended for fresh consumption or processing in agroindustries that demand high-quality starch. As a result, multi-trait and multi-environment selection in cassava breeding programs is already being practiced (Aina et al., 2009; Adjebeng-Danquah et al., 2017; Nduwumuremyi et al., 2017; Okeke et al., 2017; León et al., 2021). However, there is still a need for alternative and robust approaches for simultaneous selection for both agronomic performance and yield stability.

Olivoto et al. (2019b); Olivoto et al. (2021) have developed two methods to incorporate the weighting between mean performance and genotypic stability, which can be applied to various parametric and nonparametric approaches. The first method, mean performance and stability (MPS), calculates the weighted average of the absolute scores derived from the singular value decomposition (SVD) of the BLUPs matrix for the GEI effect. On the other hand, the second method, multi-trait mean performance and stability index (MTMPS), builds upon the concept of the multi-trait stability index (MTSI) and is based on the genotype-ideotype (Euclidean) distance using scores obtained from an exploratory factor analysis. In the MTMPS approach, the genotype with the lowest MTMPS value is considered closest to the ideotype, indicating high mean performance and stability across all evaluated traits. Therefore, the objectives of the present study were as follows: i) to assess the GEI in cassava genotypes for multiple traits, ii) to estimate the genetic parameters for seven economically important agronomic traits, iii) to identify groups of genotypes with superior productive performance, adaptability, and genotypic stability using the MPS index, iv) to select genotypes that exhibit high performance and genotypic stability across multiple traits using the MTMPS index.

## 2 Materials and methods

### 2.1 Yield trials

**Figure 1:**
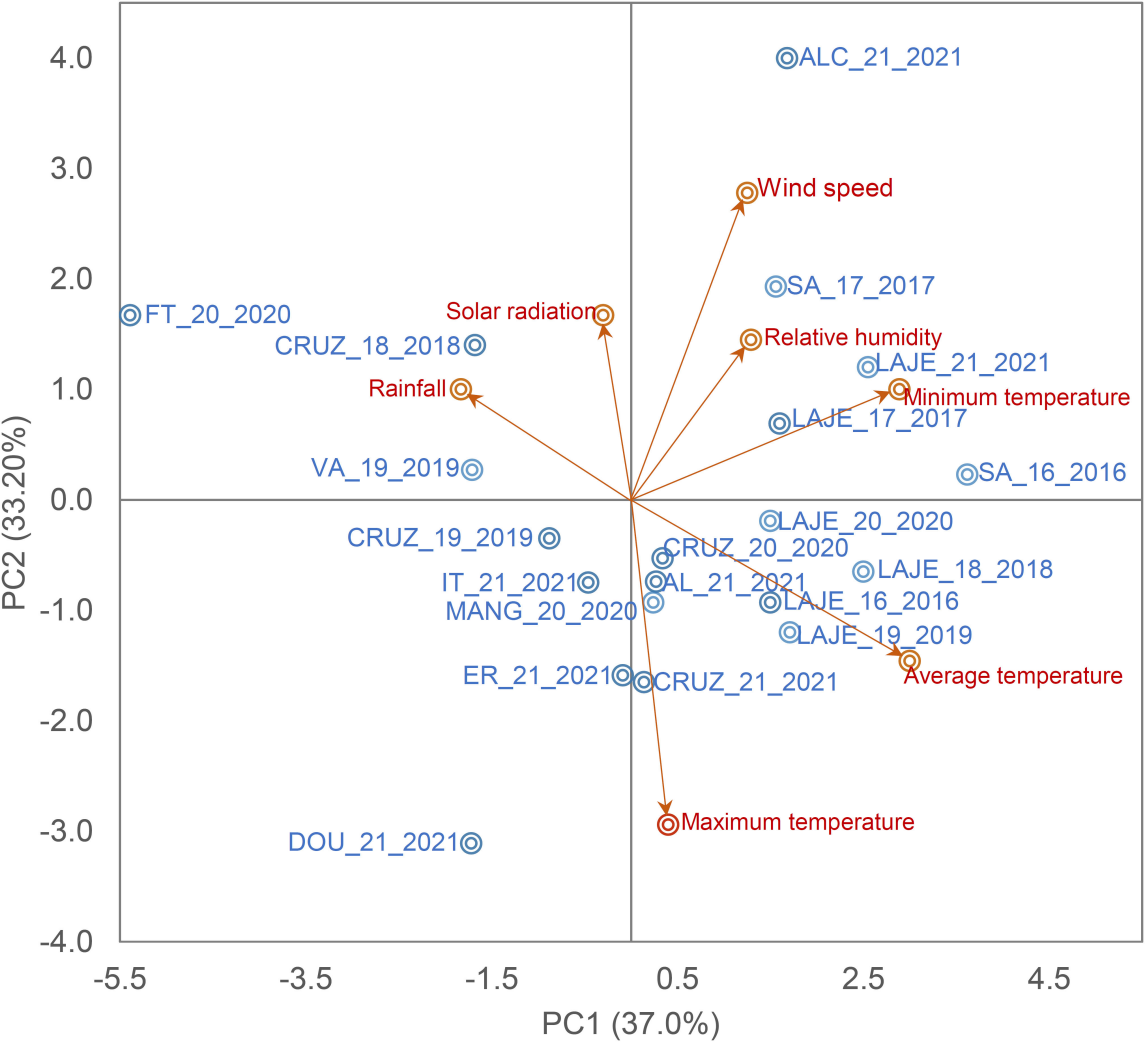
Biplot of the first two principal components of seven climate variables and the field trials. Maximum, minimum and average temperature (Tmax, °c, Tmin, °c and Tav, °c, respectively), rainfall (Rain, mm day^-1^), relative humidity (Rh, %), Wind speed (W/speed, ms^-1^) and solar radiation (Sol/rad, MJ/m^-2^ day^-1^).

The METs conducted by the cassava breeding program at Embrapa Mandioca e Fruticultura in Cruz das Almas, Bahia, Brazil (12°40’19” S, 39°06’22” W, altitude of 226 m), encompassed a total of 11 AYT and 36 UYT trials across diverse agricultural potential environments (**Table S1**). These trials were carried out over sixteen locations with every combination of year and location being regarded as an individual environment. In sum, the trials spanned 47 distinct environments. The field trials adhered to the local production system, commencing at the onset of the rainy season and continued under dryland conditions from 2016 to 2021 (**Figure 1**). The majority of these environments exhibited a tropical hot and humid climate, varying between Tropical Humid Savanna (Aw) and Tropical Monsoon (Am). The annual average precipitation was 1000 mm between April and August, coupled with an average annual temperature of 25.5°C ± 4.5°C.

A total of 22 genotypes underwent evaluation, including six clones in the final stage gate form the Embraoa breeding program (BR11-24-156, BR11-34-41, BR11-34-45, BR11-34-64, BR11-34-69, and BR12-107-002), along with 10 improved varieties released for the target area (BRS Caipira, BRS Dourada, BRS Formosa, BRS Gema de ovo, BRS Kiriris, BRS Mulatinha, BRS Novo Horizonte, BRS Poti Branca, BRS Tapioqueira, and BRS Verdinha), and six landraces used by the local farmers (Cigana Preta, Correntão, Corrente, Eucalipto, IAC-90, and Vassoura Preta).

The soil preparation for cultivation followed conventional practices, including desiccation of spontaneous plants, plowing, and harrowing to incorporate crop residues. Planting furrows were then created using a cassava planter, and fertilization was performed based on soil analysis. Manual planting was carried out horizontally in the furrows using standard cassava stems obtained from 12-month-old plants that were free of pests and diseases.

For experimental design, the trials were organized following a randomized complete block design with three replications. Each plot comprised four rows, accommodating 25 plants per row, spaced at 0.90 m between rows and 0.80 m between individual plants. Fertilization and post-planting treatments adhered to the recommended practices for cassava cultivation (as outlined by Souza et al., 2006).

### 2.2 Traits assessed

At 12 months after planting, seven agronomic traits were measured: 1) fresh root yield (FRY) represents the total weight of all the roots in the plot and is measured in tons per hectare (t ha^-1^), 2) shoot yield (ShY) represents the weight of the aboveground parts of all the plants in the plot, including stems, leaves, and petioles, also converted in tons per hectare (t ha^-1^), 3) dry matter content of the roots (DMC), determined in % using the specific gravimetric method (Kawano et al., 1987), 4) dry root yield (DRY), assessed by multiplying the FRY by the DMC and represents the weight of dry roots (t ha^-1^), 5) harvest index (HI), the ratio between the fresh weight of roots and the total biomass, including both aboveground and belowground parts of the plants, expressed as a percentage (%), 6) plant archictetucture (PIA), evaluated using a 1-5 rating scale, where: 1 excellent architecture (no branching or erect stems), 2 good architecture (branching above 1.60 m or low branching with at least 1.6 m of erect stems), 3 means moderate architecture (branching above 1.20 m or low branching with at least 1.2 m of erect stems), 4 poor architecture (branching above 0.80 m or low branching with at least 0.80 m of erect stems), and 5 very poor architecture (highly branched clones with less than 0.80 m of erect stems), 7) plant height (PH), measured using a graduated scale from the soil level to the plant meristem and is expressed in meters (m).

### 2.3 Individual and joint statistical analysis

After conducting the individual analysis of variance and ensuring the homogeneity of residual variances using the Shapiro-Wilk test, the joint analysis of variance was performed. A deviance analysis was performed considering the following mixed-effect model: $Y_{ij}=µ+\;\alpha_{i}+\tau_{j}+{\left(\alpha \;\tau \right)}_{ij}+y_{jk}+\epsilon_{ijk}$ , where $Y_{ij}$ is the response variable, µ is the general mean, $\alpha_{i}$ is the main effect of the i-th genotype (random, $\alpha_{i}∼\text{ N}(0,\;\sigma_{ɡ}^{2}$ ), $\tau_{j}\;$ is the main effect of the j-th environment (random, $\tau_{j}∼\text{ N}(0,\;\sigma_{e}^{2}$ ), ${\left(\alpha \;\tau \right)}_{ij}\;$ is the effect of the interaction between the i-th genotype and the j-th environment (random, ${\left(\alpha \;\tau \right)}_{ij}∼\text{ N}(0,\;\sigma_{ɡxe}^{2}$ ), $y_{jk}$ is the fixed effect of the k-th block within the j-th environment (i = 1, 2,…, g; j = 1, 2,…, e; k = 1, 2,…, b), and $\epsilon_{ijk}$ is the random error associated with the model with $\epsilon_{ijk}∼\text{ N}\left(0,\;\sigma_{\varepsilon }^{2}\right)$ .

The variance components were obtained using the restricted maximum likelihood (REML) using the expectation-maximization algorithm (Dempster et al., 1977), and the significance of the random effects was evaluated using the likelihood ratio test (LRT), which compares the $-2\left(RES\right)loɡ$ of two models: one with all the random terms and another with one of the random terms omitted. This comparison is performed using a chi-square (χ²) test.

Four heritability estimates were obtained: (i) Broad-sense heritability ( $H^{2}$ ), calculated as $H^{2}=\frac{\sigma_{ɡ}^{2}}{\sigma_{ɡ}^{2}+\sigma_{ɡxe}^{2}+\sigma_{\varepsilon }^{2}}$ , where $\sigma_{\text{g}}^{2}$ is the genotypic variance, $\sigma_{\text{gxe}}^{2}\;$ is the variance of the GEI interaction, and $\sigma_{\text{ε}}^{2}$ is the residual variance. (ii) plot-based heritability ( $h_{ɡm}^{2}$ ), calculated as $h_{ɡm}^{2}=\sigma_{ɡ}^{2}/\;[\sigma_{ɡ}^{2}+\sigma_{ɡxe}^{2}/e+\sigma_{e}^{2}/\left(eb\right)]$ , where e and b are the numbers of environments and blocks, respectively. (iii) Heritability of Cullis et al. (2006) ( $H_{cullis}^{2}$ ), calculated as $H_{cullis}^{2}=1-\frac{\Delta BLUP}{2\sigma_{ɡ}^{2}}$ , where ΔBLUP is the mean standard error of the genotypic BLUPs. (iv) Heritability of Piepho and Möhring (2007) ( $H_{Piepho}^{2}$ ), calculated as $H_{Piepho}^{2}$ = $\frac{\sigma_{ɡ}^{2}}{\sigma_{ɡ}^{2}+\bar{ʋ}/2}$ , where $\bar{ʋ}$ is the mean variance of a difference of two best linear unbiased estimators (BLUE). Additionally, the obtained the: i) phenotypic variance ( $\sigma_{p}^{2}$ ) given by the formula $\sigma_{p}^{2}=\left(\sigma_{ɡ}^{2}\right)+\left(\frac{\sigma_{ɡxe}^{2}}{e}\right)+\left(\frac{\sigma_{\mathrm{\varepsilon ;}}^{2}}{eb}\right)$ , where e is the number of environments; b is the number of replications; ii) genotypic coefficient of variation estimated as $CV_{ɡ}=\left(\frac{\sqrt{\sigma_{ɡ}^{2}}}{X}\right)*100\%$ , where X is the overall mean; iii) coefficient of determination of GEI effects $r_{i}^{2}$ = $\sigma_{ɡxe}^{2}\;/\;(\sigma_{ɡ}^{2}+\;\sigma_{ɡxe}^{2}+\;\sigma_{\varepsilon }^{2}$ ); iv) residual coefficient of variation estimated as $CV_{r}=\;\left(\frac{\sqrt{\sigma_{\varepsilon }^{2}}}{X}\right)*100\%$ ; v) genotype-environment correlation calculated as $rɡe=\;\sigma_{ɡ}^{2}/\left(\sigma_{ɡ}^{2}+\sigma_{ɡxe}^{2}\right)$ ; vi) CVratio given by CVɡ/CVr .

### 2.4 Adaptability and stability analyses

The genotypes were ranked using the MPS method, which has the potential to accommodate a linear mixed-effects model structure, providing new graphical insights for simultaneous selection. Additionally, it quantifies GEI through various parametric and non-parametric stability indices, including the classic method developed by Eberhart and Russell (1966), which popularized stability analyses worldwide. However, in this study, we adapted the concept of the WAASBY index (a superiority index that allows weighting between mean performance and stability – Olivoto et al., 2019a) to account for data imbalance. WAASBY utilizes the mean of the response variable and the weighted mean of absolute scores derived from the singular value decomposition of the BLUP matrix to quantify GEI effects generated by a linear mixed-effects model (LMM). Therefore, in this study, performance and stability were quantified using the WAASB method originally proposed. Still, due to data imbalance, stability was adjusted to consider genotypic stability of the variance of nonlinear regression deviations ( $S_{di}^{2}$ ), the coefficient of determination ( $R^{2}$ ) and the root mean square error of the regression ( RMSE ), according to Yue et al. (2022).

In accordance with the aforementioned indices, genotypes exhibiting zero values for both the $S_{di}^{2}$ and RMSE were conveniently assigned a rank of 1. This rank indicated that they were the most stable genotypes up to the g-th genotype. Conversely, genotypes with slop ( $R^{2}$ ) close to 1 were considered the most responsive and well-adapted up to the g-th genotype. These highly responsive genotypes are represented by yellow bars in the MPS biplots, while the least-ranked genotypes are depicted in dark blue. In order to determine the adaptability and stability parameters, as well as to rank the genotypes, the following model was utilized: $y_{ij}=\beta_{oi}\;+\beta_{1i}\;I_{j}\;+\delta_{di}^{2}+\epsilon_{ij}\;$ , where $y_{ij}$ represents the mean of genotype i in environment j, $\beta_{oi}\;$ is the constant regression term, representing the overall mean of genotype i, $\beta_{1i}\;$ is the coefficient of the linear regression, indicating the response of the i-th genotype to variations in the j-th environment, $I_{j}$ represents the coded environmental index, $\delta_{di}^{2}\;$ corresponds to the variance of the regression deviations, $\epsilon_{ij}\;$ represents the mean experimental error.

The first step involved rescaling the agronomic performance and the stability matrix, so that they can be directly compared, using the following model: $r_{Yi}=\frac{nmax\;-\;nmin}{\;Y_{\max\;}-\;Y_{min}}\;x{\;}_{\left(Yi-Ymax\right)}\;$ + *nmax*, as well as $r_{Ei}=\;\frac{nmin-nmax\;}{E_{max}\;-\;E_{min}}\;x{\;}_{\left(Ei\;-\;Emax\right)}$ + *nmin* respectively, where $r_{Yi}$ and $r_{Ei}$ are the rescaled values for genotypic performance and stability of the i-th genotype, respectively; *nmax* and *nmin* are the new maximum and minimum values of the variables and the MPS index ( $S_{di}^{2},\;R^{2}$ and RMSE ) after the rescaling process; Yi and Ei represent the original values of the variable response and MPS index of the i-th genotype, respectively.

For the $\;R^{2}$ and the variables FRY, ShY, DMC, and DRY, higher values are considered desirable. This is reflected in the assigned values: $Y_{max}=100$ and $Y_{min}=0$ , and $E_{max}=100$ and $E_{min}=0$ . Consequently, a genotype with the highest mean and the highest $\;R^{2}$ would achieve $r_{Yi}$ and $r_{Ei}$ = 100. An exception to this pattern is applied to plant architecture trait, where smaller values are preferred to ensure suitability for mechanized planting. Hence, $Y_{max}=0$ and $Y_{min}=100$ for this particular trait. Conversely, for the $S_{di}^{2}$ and RMSE indices, lower values are sought after, as they indicate greater stability. Therefore, the environmental index was adjusted with $E_{max}=0$ and $E_{min}=100$ . These specific value selections were made in accordance with the characteristics of the response variable. Notably, in six of these variables, larger values are preferred, catering to both consumer preferences and selection criteria.

After rescaling, the best-performing genotype received a score of 100, while the worst-performing genotype obtained a score of 0. Similarly, the genotype with the best stability was assigned a value of 100, while the genotype with the poorest stability received a value of 0. Thus, a two-way matrix was created with a range spanning from 0 to 100 along its columns, aligning with the direction of the selection process.

In the second stage, the MPS index was computed using the following equation: $MPS=\;\frac{\left(rY_{i\;x\;}\theta_{\gamma }\right)+\;\left(rE_{i\;x\;}\theta_{S}\right)}{\theta_{\gamma }+\theta_{S}}$ , where MPS represents the index that weighs performance and stability for the i-th genotype, $\theta_{\gamma }$ e $\theta_{S}$ are the weights assigned to the response variable (performance) and stability, respectively. In this study, the weights were set at 65% for performance and 35% for stability at the MTMPS index. This weighting prioritized average performance over stability, as the goal was not to select varieties for release that are highly stable but lack good agronomic performance.

We also analyzed the variation in weights between performance and stability, getting 21 scenarios represented by $\theta_{\gamma }-\theta_{S}\;$ ranging from 0/100 to 100/0, in order to understand their impact. The rankings were found to change depending on the weight assigned to the response variable and stability. The rankings on the far-left side were obtained by considering only stability, while the rankings on the far-right side were based solely on performance. The rankings in between varied according to the assigned weights for performance. This flexibility allows for selection at different stages of the breeding process. To facilitate an intuitive interpretation of these scenarios, a heatmap was generated.

Next, the MTMPS was used to compute the mean performance and stability of multiple traits (Yue et al., 2022). For this stage, the concept of genotype-ideotype (Euclidean) distance using exploratory factor analysis was employed to group correlated variables into factors and compute the factorial scores for each genotype. The MTMPS index was determined using the following model: $MTMPS_{i}={\left[\sum_{j=1}^{f}{\left(F_{ij}-F_{j}\right)}^{2}\right]}^{0.5}$ , where $MTMPS_{i}$ represents the multi-trait stability index for the i-th genotype, $F_{ij}$ denotes the j-th score of the i-th genotype with ( i=1, 2,…,ɡ ) e $\left(j=1,2,\ldots ,f\right)$ , where *g* is the number of genotypes and *f* is the number of factors, respectively, and $F_{j}$ represents the j-th score of the ideotype. The genotype with the lowest MTMPS score is considered to be the closest to the ideotype and therefore exhibits high MPS for all n performance and stability variables analyzed.

Furthermore, the study calculated the selection differential for the selected genotypes, assuming a selection intensity of 30%, in order to determine the selection gain for stability and performance. This calculation was done based on the genotypes selected by the MTMPS index, using the following formula: $\Delta S\%=\left(X_{S}-X_{0}\right)/\;X_{0}\;x\;100$ , where $X_{S}$ and $X_{0}$ represents the values of the selected genotypes and population mean, respectively.

The analyses were conducted using the metan package v1.18.0, in R software version 4.2.0 (R Core Team, 2022), employing the functions gamem_met(), mps(), and mtmps() (Olivoto and Lúcio, 2020). The heritability was determined using the H2cal() function from the inti package.

### 2.5 Exploratory factor analysis

Exploratory factor analysis was employed to group correlated traits and calculate genotypic factor scores. The model used for analysis was X=μ+Lf+ϵ , where X=px1 vector of rescaled observations, μ=px1 vector of standardized means, L=pxf matrix of factor loadings, f=px1 vector of common factors, and ϵ=px1 the vector of residuals. p and f is the number of traits and common factors retained, respectively. Eigenvalues and eigenvectors were derived from the genetic correlation matrix, which were then subjected to varimax rotation (Kaiser, 1958) to obtain the final loadings used in calculating genotypic scores. This calculation is represented as: $F=Z\;{\left(A^{T}R^{-1}\right)}^{T}$ , where F=qxf is matrix with factor scores; Z=qxp is a matrix with rescaled means; A=pxf is a matrix of canonical loadings; R=pxp is a matrix representing the correlation of MPS values; q, f and p represent genotypes, traits, and retained factors, respectively.

Subsequently, a network analysis was performed to assess the correlations between the traits. Pearson’s test was utilized to estimate these correlations, employing the correlate function from the corrr package v 0.4.4 (Kuhn et al., 2020) in R software version 4.2.0 (R Core Team, 2022). This analysis aimed to provide insights into the interrelationships among the traits under investigation.

## 3 Results

### 3.1 Analysis of variance of the agronomic data

The LRT test revealed significant effects (p<0.001) for genotypes, environments, and genotype-environment interaction (GEI). These findings highlight the presence of genetic variability, leading to changes in genotype classification across various agronomic attributes analyzed. Furthermore, it underscores the importance of evaluating genotype performance while considering environmental variations (**Table 1**). Notably, the GEI of the crossover type was identified for the evaluated traits, introducing bias in predicting genetic advancements and reducing selection gains (**Figure S1**).

**Table 1:** Summary of the joint maximum likelihood ratio test for fresh root yield (FRY), shoot yield (ShY), dry root yield (DRY), dry matter content (DMC), plant architecture (PIA), harvest index (HI) and plant height (PH) of 22 cassava genotypes evaluated in 47 environments.

Source of variation^1^	Characteristics						
	FRY	ShY	DRY	DMC	PIA	HI	PH
Genotypes (G)	193.90^***^	255.51^***^	192.40^***^	483.48^***^	200.11^***^	332.18^***^	362.77^***^
Environments (E)	176.85^***^	209.64^***^	195.92^***^	190.93^***^	79.70^***^	259.0^***^	245.60^***^
Rep : Env	53.46^***^	57.97^***^	44.08^***^	47.43^***^	9.19^**^	7.62^**^	22.46^***^
GEI	444.27^***^	384.56^***^	412.43^***^	306.93^***^	240.94^***^	444.70^***^	291.96^***^
$\hat{r}=\hat{ɡɡ}$	0.99	0.99	0.99	0.99	0.99	0.98	0.99
Overall average	24.22	20.94	7.64	35.58	2.44	54.04	2.24

^1^Variance due to genotype, environment and GEI interaction, ** and *** significant at p<0.01% and p<0.001% probability, respectively by F test; ${\hat{r}}_{\hat{ɡɡ}}$ : selective accuracy.

For the FRY trait, genotypes BR11-34-69 and BR11-34-41 demonstrated the highest FRY (>31.05 t ha^-1^), representing a 30% superiority compared to the average of evaluated genotypes (23.80 t ha^-1^). In contrast, genotypes BR11-34-69, BRS Formosa, and Vassoura Preta exhibited the highest HI (>60.0%) in relation to the overall average of 54.33% (**Table S2**). It’s important to note that each analyzed variable responded differently across environments. For instance, the most favorable environments for FRY and HI were 2020.ERU.UFV (with an average of 38.78 t ha^-1^) and 2019.ERU.GA (with an average of 72.01%), respectively (**Figure S2**).

Genotypes BRS Novo Horizonte, BR11.34.64, BR11.34.45, BRS Mulatinha, and Correntão exhibited ShY above 23.0 t ha^-1^ (>21% higher than the overall average of evaluated clones). Environmental influence was also significant, ranging from 10.39 t ha^-1^ in the 2016.ERU.SA environment to 50.28 t ha^-1^ in 2020.ERU.UFV. High DMC contents were observed in genotypes BRS Novo Horizonte, BRS Mulatinha, and BR12-107-002 (>37%). Although there was relatively smaller environmental variation for this trait (ranging from 30.79% in the 2021.ERU.AL environment to 38.26% in the 2019.ERU.NR environment). The ranking of genotypes based on the DRY trait highlighted BRS Novo Horizonte and BR11-34-69 as the most productive (>9.0 t ha^-1^), with only BRS Novo Horizonte exhibiting high DMC. The DRY trait varied from 2.55 t ha^-1^ in the 2021.ERU.UFGD environment to 12.56 t ha^-1^ in the 2021.ERU.NH4 environment, with an average range of genotypes for this trait across environments being 4.39 to 9.36 t ha^-1^.

For the PIA trait, genotypes IAC-90, BR11-34-64, BR11-34-69, and BR12-107-002 exhibited scores below 2, indicating good plant architecture suitable for mechanized planting and harvesting. There was significant variation in this characteristic, ranging from 4.70 (genotype BRS Verdinha) to 1.29 (genotype BR12^-1^07-002). Regarding the PH trait, genotypes BRS Tapioqueira, BR11-34-64, BR11-34-69, and BRS Poti Branca displayed the tallest plants (>2.4m), representing an 11% superiority compared to the average of tested genotypes. The average plant height across environments ranged from 1.57m (in the 2021.ERU.UFRB environment) to 3.03m (in the 2020.ERU.UFV environment).

Despite cassava being primarily propagated vegetatively, the GEI strongly influences phenotype expression. This can be observed in the distribution of means for the evaluated traits, which show high variation both between and within environments. Notably, variables like FRY and ShY exhibited significant variation, while DMC showed greater consistency across different environments. The PH trait also demonstrated consistent variation across environments.

### 3.2 Variance components and genetic parameters

During the breeding process, it is essential to gather genetic parameters to guide genotype selection and enhance the reliability of our breeding program. In our study, we observed substantial genetic ad GEI variance contributions to the total phenotypic variation across all traits. This highlights the varying behavior of genotypes in response to different environmental conditions. Consequently, it becomes important to take into account stability parameters and productive adaptability when making genotype selections. The GEI component exhibited a range from 20.67% for DMC to 38.54% for FRY, with an overall $\sigma_{ɡxe}^{2}$ effect exceeding 30% for traits such as FRY, ShY, DRY, and HI. Except for DMC, a significant proportion of the phenotypic variance was attributed to residual variance ( $\sigma_{e}^{2}$ )) for the other traits, spanning from 26.43% for HI to 31.44% for DRY (**Table 2**). Genetic variability also played a substantial role in the total phenotypic variance, notably for DMC and PH, where $\sigma_{ɡ}^{2}\;$ accounted for 55.87% and 48.75%, respectively. $H^{2}\;$ values ranged from low for FRY, ShY, HI, and DRY (between 0.30 and 0.40) to moderate for PIA, PH, and DMC (ranging from 0.41 to 0.57) (**Table 2**). Conversely, estimates such as $H_{cullis}^{2}$ , $h_{mɡ}^{2}$ , and $H_{Piepho}^{2}$ were high for all traits, ranging from 0.83 for ShY to 0.93 for DMC. The genotypic coefficient of variation ( CVɡ ) exhibited a wide range, from 4.28% for DMC to 31.40% for PIA, showcasing substantial genetic diversity among the genotypes. On the other hand, the residual coefficient of variation ( CVr ) ranged from 2.77% for DMC to 28.20% for PIA. Furthermore, the ratio between the genotypic and residual coefficient of variation ( CVratio ) showed values greater than 1 for all traits except ShY and DRY. Lastly, the magnitude of the correlation of GEI interaction effects ( rɡe ) was moderate, ranging from 0.42 to 0.56.

**Table 2:** Estimates of variance components and heritability for seven agronomic traits of 22 cassava genotypes evaluated in 47 environments.

Parameters	FRY	ShY	DMC	DRY	PH	HI	PIA
$\sigma_{p}^{2}$	54.8	49.20	4.15	5.50	0.09	73.10	1.41
$H^{2}$	0.32	0.30	0.57	0.31	0.50	0.40	0.42
$h_{mɡ}^{2}$	0.94	0.94	0.98	0.94	0.97	0.96	0.97
$H_{Cullis}^{2}$	0.87	0.83	0.93	0.86	0.90	0.91	0.88
$H_{Piepho}^{2}$	0.92	0.91	0.97	0.90	0.95	0.94	0.90
r2	0.38	0.37	0.20	0.37	0.23	0.32	0.24
rɡe	0.56	0.53	0.46	0.54	0.46	0.55	0.42
$\sigma_{ɡxe}^{2}$	20.55	17.91	0.86	2.08	0.02	23.36	0.35
CVɡ	17.20	18.60	4.28	17.10	9.37	10.10	31.40
CVr	16.70	19.0	2.77	17.20	7.02	8.13	28.20
CV ratio	1.03	0.97	1.54	0.99	1.34	1.24	1.11

$\sigma_{p}^{2}$ , phenotypic variance; $H^{2}$ , broad sense heritability; $h_{mɡ}^{2}$ , plot-based heritability; $H_{Cullis}^{2}$ and $H_{Piepho}^{2}$ , Cullis and Piepho heritability, respectively; r2 , coefficient of determination of the GEI interaction; rɡe , correlation of GEI interaction; $\sigma_{ɡxe}^{2}$ , GEI variance; CVɡ , genotypic coefficient of variation (%); CVr , residual coefficient of variation (%) and CV ratio , ratio between the coefficient of genotypic and residual variation (%); FRY, fresh root yield; ShY, shoot yield; DMC, root dry matter content; DRY, dry root yield; PH, plant height; HI, harvest index; PIA, plant architecture.

### 3.3 Exploratory factor analysis

In the factor analysis, we retained three principal components (PC) to explain genetic variations in the dataset. These components explained a cumulative variance of 92.20% for $S_{di}^{2}$ (stability), 86.60% for *R* ^2^, and 85.50% for RMSE (**Table 3**). After varimax rotation, we calculated the average communality (ɧ), representing the proportion of variance shared among factors. The average ɧ values were 0.92 for $S_{di}^{2}$ , 0.86 for *R* ^2^, and 0.85 for RMSE. For the parameter $S_{di}^{2}$ the range of communality was 0.76 ≤ ɧ ≥ 0.98, while for *R* ^2^ it ranged from 0.75 ≤ ɧ ≥ 0.97, and RMSE was 0.68 ≤ ɧ ≥ 0.95 for the traits DMC and DRY.

**Table 3:** Factor loadings, commonality, eigenvalues and explained variance after varimax rotation, obtained in the factor analysis for the parameters $S_{di}^{2}$ , (regression deviations), *R* ^2^ (regression determination coefficient) and RMSE (square root mean square error) of the regression with a weight of 65% for performance and 35% for stability, respectively.

Trait	Parameters											
	$S_{di}^{2}$				*R* ^2^				RMSE			
	FA1	FA2	FA3	Com	FA1	FA2	FA3	Com	FA1	FA2	FA3	Com
Fresh root yield	**-0.70**	**-0.65**	0.03	0.92	**-0.96**	0.12	0.15	0.96	**-0.95**	-0.10	0.01	0.93
Shoot yield	**-0.96**	0.01	-0.20	0.95	-0.37	**0.81**	-0.24	0.86	-0.21	**-0.72**	-0.5	0.83
Harvest index	0.10	**-0.97**	0.11	0.96	-**0.45**	**-0.77**	0.29	0.89	**-0.61**	**0.68**	0.15	0.87
Plant height	**-0.90**	0.12	0.34	0.96	-0.32	**0.84**	0.12	0.84	-0.23	**-0.86**	0.36	0.94
Dry matter content	**-0.64**	-0.15	**-0.57**	0.76	-0.14	0.24	**-0.83**	0.75	-0.33	-0.15	**-0.74**	0.68
Dry root yield	**-0.78**	**-0.59**	-0.17	0.98	**-0.94**	0.18	-0.05	0.97	**-0.96**	-0.12	-0.09	0.95
Plant architecture	-0.05	-0.12	**0.95**	0.92	-0.23	0.04	**0.83**	0.76	-0.29	-0.16	**0.81**	0.78
Eigenvalues	3.64	1.55	1.27	–	2.85	2.13	1.08	–	2.62	1.84	1.55	–
Variance	52.0	22.10	18.10	–	40.70	30.40	15.50	–	37.40	26.30	22.10	–
Accumulated %	52.0	74.10	92.20	–	40.70	71.10	86.60	–	37.40	63.70	85.80	–

FA, retained factor, values in bold indicate variables grouped within each factor, *Com-Communality.

Agronomic traits were categorized based on stability parameters: $S_{di}^{2}$ , *R* ^2^, and RMSE . For $S_{di}^{2}$ , the first factor strongly associated with ShY (-0.96) and PH (-0.90) – associated with planting density –, and moderately with DRY (-0.78), DMC (-0.64), and FRY (-0.70) – related to root yield. The second factor was strongly associated to HI (-0.97) and moderately to FRY (-0.65) and DRY (-0.59). The third factor encompassed PIA (0.95) and DMC (-0.57) with opposite loadings.

Regarding *R* ^2^ and RMSE parameters, the first factor grouped FRY (-0.96 and -0.95) and DRY (-0.94 and -0.96) with high and consistent loadings. HI (-0.45 and -0.61) exhibited loadings of moderate magnitude. The second factor grouped PH with opposing high loadings (0.84 and -0.86), along with ShY (0.81 and -0.72) and HI (-0.77 and 0.68) showing opposing loadings of moderate magnitude. Finally, the third factor included PIA with high loadings in the same direction (0.83 and 0.81), while DMC showed loadings in different directions (-0.83 and 0.74).

### 3.4 Performance and stability of the primary selection traits (FRY, ShY, DMC and DRY) via MPS for the parameters $S_{di}^{2}$ , *R* ^2^ and RMSE

**Figure 2:**
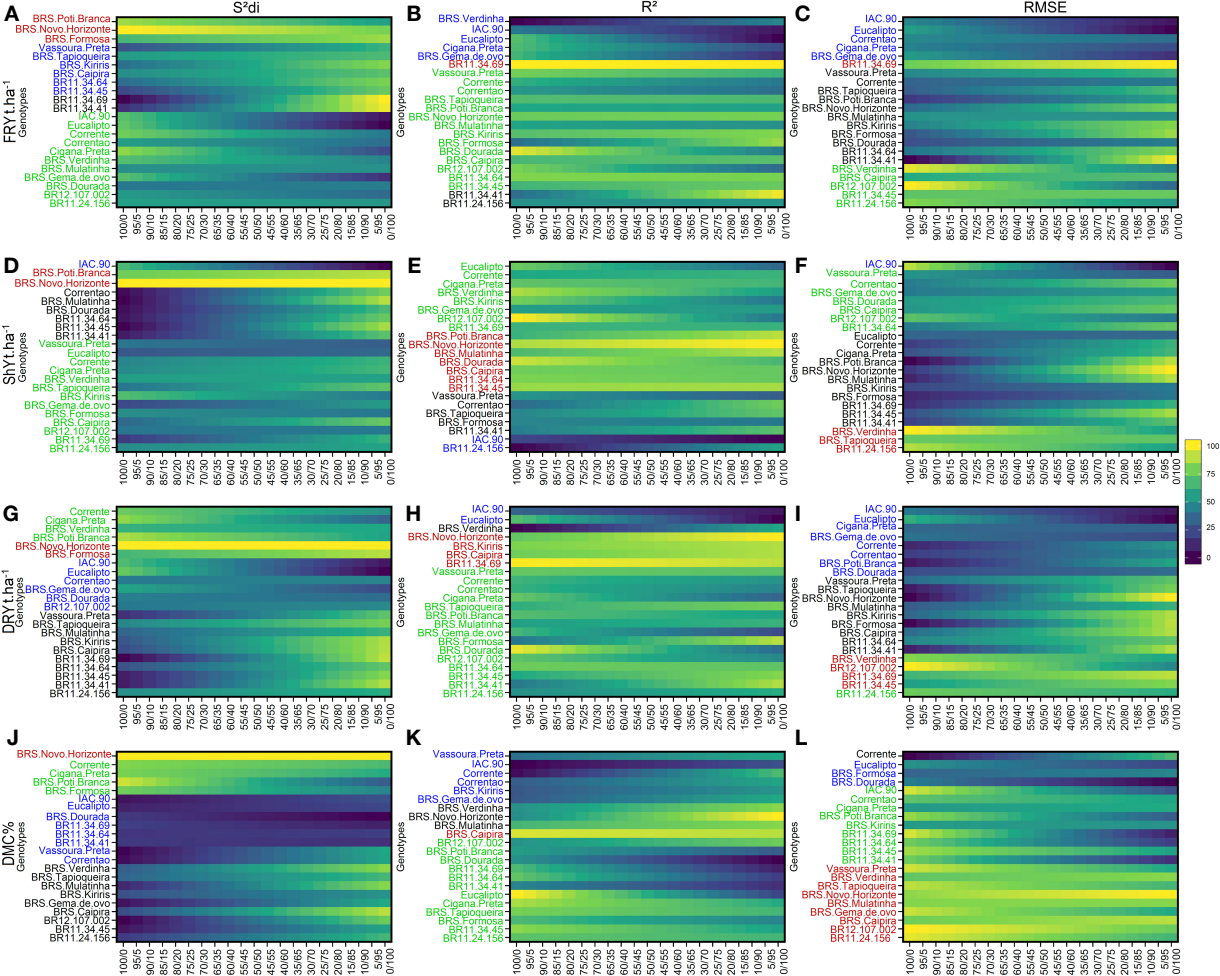
Rank of cassava genotypes considering the mean performance and stability (MPS) index with variation of economic weights from 0 to 100% for parameters $S_{di}^{2}$ (regression deviations), *R* ^2^ (coefficient of determination) and RMSE (root square of the mean squared error of the regression) of the stability model of Eberhart and Russell (1966). The color gradient (from 0 to 100), ranging from dark blue to yellow, represents the variation from unproductive to highly productive, respectively, for the following primary traits: **(A–C)** for fresh root yield (FRY, t ha^-1^); **(D–F)** for shoot yield (ShY, t ha^-1^); **(G–I)** for dry root yield (DRY, t ha^-1^) **(J, K)**, and **(J–L)** for dry matter content (DMC, %). The most-left ranks were obtained considering the stability only, while the right-ranks represents genotypes with the highest agronomic performance. The scenarios between the extremes exhibit a 5% variation in the classification.

We selected the parameters $S_{di}^{2}$ , *R* ^2^ and RMSE for their capacity to distinguish the effects of heterogeneity between genotypes/environments. These parameters were employed to assess how genotype rankings for each agronomic trait varied based on both their performance and stability. The plots on the left represent genotype ranking solely based on stability, while the plots on the right represent ranking based solely on agronomic performance. The weighting between stability and agronomic performance was varied in 5% increments. Four main groups of genotypes were formed based on the similarity of stability and agronomic performance, as described below: Group 1: Highly productive and stable genotypes (indicated in red); Group 2: Stable but low productive genotypes (indicated in green); Group 3: Productive but unstable genotypes (indicated in black); and Group 4: Low productive and unstable genotypes (indicated in blue) (**Figure 2**).

Among the genotypes, BRS Poti Branca and BRS Novo Horizonte were assigned to Group 1 due to their high stability for FRY and ShY, as indicated by the $S_{di}^{2}\;$ parameter. However, when considering the *R* ^2^ and RMSE parameters, only the genotype BR11-34-69 was identified as more stable for FRY. For ShY, genotypes such as BRS Poti Branca, BRS Novo Horizonte, BRS Mulatinha, BRS Dourada, BRS Caipira, BR11-34-45, BR11-34-64, and BR11-24-156 were classified as stable and high-performing, showing consistent performance across different environments (**Figure 2**).

Group 2 included a larger number of genotypes (approximately 13 genotypes) based on the $S_{di}^{2}\;$ parameter, and around 8 genotypes based on the *R* ^2^ parameter. This indicates the challenge in selecting genotypes that are both stable and high performing, with a noticeable variability within the group. A smaller subset of genotypes (approximately 5) was grouped based on the RMSE parameter. Among these, the genotype BR12-107-002 was common for all three parameters, while BR11-24-156 was common for $S_{di}^{2}$ , Corrente for *R* ^2^, and BRS Caipira for RMSE , in both ShY and FRY traits. In Group 3, only the genotype BR11-34-41 was grouped based on all three parameters for both FRY and ShY. The RMSE parameter included the largest number of genotypes within Group 3, with 11 clones being grouped. Lastly, in Group 4, the genotype IAC-90 was grouped based on all three parameters, except for $S_{di}^{2}\;$ for FRY, which identified Vassoura Preta as an unproductive and unstable genotype in these evaluation regions.

For the DRY and DMC traits, Group 1 consisted of genotypes that had both high performance and stability, and it grouped the smallest number of genotypes. For DRY and DMC, the genotype BRS Novo Horizonte was allocated to Group 1 based on the $S_{di}^{2}\;$ parameter, while BRS Caipira was classified as Group 1 based on *R* ^2^. BR12-107-002 and BRS Verdinha were grouped in Group 1 for the RMSE parameter.

For DRY, the largest number of genotypes was allocated to Group 2, particularly based on the $S_{di}^{2}\;$ parameter. The RMSE parameter only grouped the BR11-24-156 genotype, which was already grouped based on the previous parameter. In contrast, for the DMC trait, a greater number of genotypes were grouped in Group 2. The genotypes BRS Poti Branca, Cigana Preta, BR11-34-41, BR11-34-45, BR11-34-64, and BR11-34-69 were common for both the *R* ^2^ and RMSE parameters. Additionally, the $S_{di}^{2}\;$ parameter included three genotypes (Corrente, Cigana Preta, and BRS Poti Branca) that were common to both the DRY and DMC traits within Group 2.

Group 3 comprised nine genotypes grouped based on the $S_{di}^{2}\;$ parameter, with BR11-34-45 and BR11-24-156 being common for both the DRY and DMC traits. Different genotypes were grouped based on the *R* ^2^ and RMSE parameters for these two traits. In Group 4, the genotypes IAC-90 and Eucalipto were grouped based on all three parameters, while BRS Dourada was grouped based on only two parameters ( $S_{di}^{2}\;$ and RMSE ) for the DRY. For the DMC trait, the genotypes IAC-90, Vassoura Preta, and Correntão were commonly grouped based on the $S_{di}^{2}\;$ and *R* ^2^ parameters, whereas Eucalipto and BRS Dourada were grouped based on the $S_{di}^{2}\;$ and RMSE parameters.

### 3.5 Performance and stability of the secondary traits (HI, PH and PIA) via MPS for $S_{di}^{2}$ , *R* ^2^ and RMSE parameters

**Figure 3:**
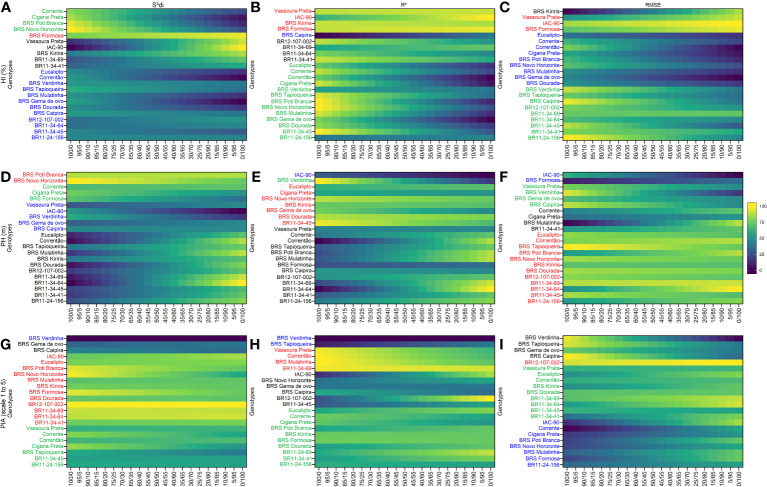
Rank of cassava genotypes considering the mean performance and stability (MPS) index with variation of economic weights from 0 to 100% for parameters $S_{di}^{2}$ (regression deviations), *R* ^2^ (coefficient of determination) and RMSE (root square of the mean squared error of the regression) of the stability model of Eberhart and Russell (1966). The color gradient (from 0 to 100), ranging from dark blue to yellow, represents the variation from unproductive to highly productive, respectively, for the following primary traits: **(A–C)** for harvest index (HI, %); **(D–F)** for plant height (PH, m); **(G–I)** for plant architecture (PIA, scale 1 to 5). The most-left ranks were obtained considering the stability only, while the right-ranks represents genotypes with the highest agronomic performance. The scenarios between the extremes exhibit a 5% variation in the classification.

Using the $S_{di}^{2}$ , *R* ^2^ and RMSE parameters, four distinct and uncorrelated groups were identified for secondary traits (**Figure 3**). BRS Formosa stood out in Group 1 for the HI trait, showing high stability and consistent agronomic performance across all three stability parameters. Vassoura Preta and IAC-90 were also grouped in Group 1 based on the *R* ^2^ and RMSE parameters. Group 2 comprised stable but low-performing HI genotypes, including checks such as Corrente, Cigana Preta, BRS Poti Branca, and BRS Novo Horizonte based on the $S_{di}^{2}$ parameter. The *R* ^2^ and RMSE parameters grouped genotypes such as BR11-34-45, BR11-24-156, BRS Verdinha, and BRS Tapioqueira in Group 2. Group 3 featured productive but unstable HI genotypes, with BRS Kiriris grouped by the $S_{di}^{2}\;$ and RMSE parameters, and genotypes BR12-107-002, BR11-34-69, BR11-34-64, and BR11-34-41 grouped by the *R* ^2^ parameter. Group 4 included a larger number of genotypes with high variability within the group, as indicated by the $S_{di}^{2}$ parameter (12 genotypes) and the RMSE parameter (9 genotypes), while the *R* ^2^ parameter only grouped BRS Caipira.

For the PH trait, Group 1 consisted of 12 clones grouped based on the RMSE parameter, including genotypes BR11-34-64, BR11-34-69, BR11-34-45, BR11-24-156, and BR12-107-002, as well as BRS Novo Horizonte, which was also grouped by the $S_{di}^{2}$ and *R* ^2^ parameters. Group 3 encompassed a significant number of genotypes (13 for $S_{di}^{2}$ and 12 for *R* ^2^), with common genotypes such as BR11-34-64, BR11-34-69, BR11-34-41, BR11-24-156, and BR12-107-002. In contrast, Group 4 consisted of the smallest number of genotypes, with IAC-90 being grouped in all three parameters.

For the PIA trait, BR12-107-002 was placed in Group 1, primarily based on the parameters $S_{di}^{2}\;$ and RMSE . Additionally, the genotype BR11-34-64 was grouped with BR12-107-002 based on $S_{di}^{2}$ and *R* ^2^ parameters. These genotypes demonstrated high performance and consistent stability for PIA, regardless of the stability-to-performance ratio (0/100). The rank order of these genotypes remained unchanged regardless of the variation in ranking criteria. Interestingly, the $S_{di}^{2}\;$ parameter played a significant role in grouping 45% of the genotypes in Group 1, which included four newly identified genotypes (BR12-107-022, BR11-34-69, BR11-34-64, and BR11-34-41). In Group 4, the genotype BRS Verdinha was categorized as having low performance and instability for PIA, based on the $S_{di}^{2}\;$ and *R* ^2^ parameters. No other clones met the stability criteria for PIA in Group 4. Notably, the classification of genotypes in Group 4 did not exhibit significant variations with respect to different weight ratios between stability and agronomic performance, particularly for the $S_{di}^{2}\;$ parameter.

### 3.6 Truncated selection of cassava genotypes based on the MPS index

The MPS index selection differential for the $S_{di}^{2}\;$ parameter varied from -4.06% (DMC) to 29.8% (FRY). Most traits showed similar differentials, ranging from 21.3% to 29.8%, except for ShY and PH, which had different values of 15.6% and 18.1%respectively (**Table 4**). The gains of the MPS index for the *R* ^2^ parameter exhibited greater variability, ranging from -3.08% (DMC) to 33.0% (FRY). Gains exceeding 25% were observed only for the DRY and FRY traits. Similar variability was observed for the MPS gains in the RMSE parameter, which ranged from 12.3% (DMC) to 29.1% (DRY). Consequently, the selection differential for the MPS index, considering the $S_{di}^{2}$ , *R* ^2^, and RMSE parameters, resulted in positive gains across all variables, demonstrating the model’s efficacy in capturing improvements in both performance and stability. The only exception was the DMC trait, where a reduction of 4.0% and 3.0% was observed in the MPS index for the $S_{di}^{2}\;$ and $R^{2}$ parameters, respectively.

**Table 4:** Selection differential for the Mean Performance and Stability (MPS) stability index of cassava variables using the parameters $S_{di}^{2}$ (regression deviations) *R* ^2^ (regression determination coefficient) and RMSE (root mean square error) regression of the Eberhart and Russell, 1966 model, with an economic weight of 65% for performance and 35% for stability, respectively.

Traits	Factor	$S_{di}^{2}$ /FA				Factor	*R* ^2^ FA				Factor	RMSE /FA			
		Xo	Xs	SD	SD%		Xo	Xs	SD	SD%		Xo	Xs	SD	SD%
Fresh root yield	FA1	54.3	70.4	16.2	29.8	FA1	57.3	76.2	18.9	33.0	FA1	52.7	67.5	14.8	28.2
Shoot yield	FA1	52.3	60.5	8.17	15.6	FA2	61.6	73.6	11.9	19.3	FA2	54.0	61.7	7.65	14.2
Plant height	FA1	55.4	65.4	10.0	18.1	FA2	59.3	69.3	10.0	16.9	FA2	65.4	77.0	11.6	17.7
Dry matter content	FA1	44.3	42.3	-2.02	-4.6	FA3	52.9	51.3	-1.62	-3.6	FA3	58.9	66.1	7.22	12.3
Dry root yield	FA1	55.6	69.7	14,1	25.4	FA1	61.3	78.2	16.9	27.6	FA1	52.1	67.2	15.2	29.1
Harvest index	FA2	44.9	57.5	12.5	27.9	FA2	54.1	61.4	7.30	13.5	FA2	50.2	58.6	8.47	16.9
Plant architecture	FA3	66.7	81.0	14.2	21.3	FA3	61.9	70.9	9.05	14.6	FA3	59.9	69.1	9.12	15.2

Xo , original mean; Xs , mean of selected genotypes; SD , selection differential; SD% , selection differential in percentage.

### 3.7 Correlations between agronomic traits

**Figure 4:**
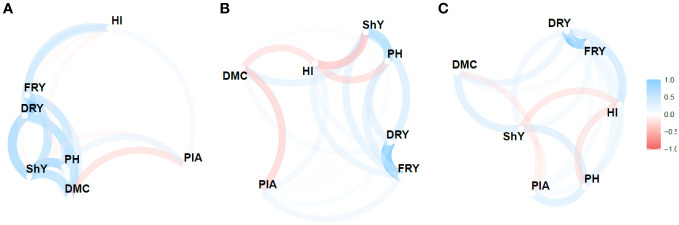
Pearson’s correlation network between the agronomic variables for the Mean Performance and Stability (MPS) index for the parameters: **(A)** $S_{di}^{2}$ (regression deviations), **(B)** *R* ^2^ (regression determination coefficient) and **(C)** RMSE (root mean square regression error) of the model by Eberhart and Russell, 1966, with an economic weight of 65% for agronomic performance and 35% for stability, respectively. The thickness of the lines represents the magnitude of the correlations, while the blue and red colors represent positive and negative correlations, respectively.

A network diagram (**Figures 4**, **S3**) was used to visualize the relationship between the MPS indices based on the stability parameters $S_{di}^{2}$ , *R* ^2^ and RMSE along with the calculation of Pearson’s correlation coefficient to assess these relationships. The analysis revealed considerable variability in both direction and magnitude of the estimates of the relationship between agronomic traits. Moreover, the three stability parameters differed in terms of the number of identified correlations. $S_{di}^{2}$ , *R* ^2^ and RMSE detected 47.61%, 33.30%, and 28.57% of the correlations as significant and of high magnitude, respectively.

Despite the variability, certain pairs of traits showed consistent correlations across the $S_{di}^{2}$ , *R* ^2^ and RMSE parameters. Notably, FRY × DRY displayed a highly positive correlation (>0.95), indicating a strong relationship between these traits. Similarly, ShY × PH exhibited a relatively strong correlation (>0.46). However, the negative correlations observed between ShY × HI were only significant for the *R* ^2^ parameter (-0.50), albeit with moderate magnitude. Additionally, modest negative correlations were found between DMC × PIA for all three parameters, ranging from -0.26 to -0.43. Among these correlations, only the *R* ^2^ parameter yielded significant results.

The network analysis revealed distinct groupings of primary agronomic traits (FRY, ShY, and DRY) and secondary traits (PH and PIA). These groupings often exhibited negative correlations, such as DMC × PIA and HI × ShY, which pose challenges in simultaneous selection of genotypes based on multi-trait indices. However, trail analysis demonstrated that a significant portion of the variation in FRY could be explained by the direct effects of two primary traits: DRY (0.97) and ShY (0.50). These traits exhibited *R* ^2^values exceeding 0.98 and showed a low residual correlation (0.16). Furthermore, they had variance inflation factor values below 10, indicating low multicollinearity. Consequently, these traits hold value for indirect selection and the utilization of multi-trait indices (e.g., MTMPS) that contribute significantly to the main trait, fresh root yield (**Table S3**).

### 3.8 Multi-trait selection for performance and stability based on the MTMPS index

The MTMPS index provides insights into the selection differential, selection gains, and heritability of selected across various agronomic traits (**Table 5**). Overall, the MTMPS index gains for the *R* ^2^ and RMSE parameters showed a similar pattern. In terms of *R* ^2^ parameter, most traits exhibited gains ranging from 2.62% (HI) to 18.90% (FRY), except for PIA, which showed a reduction of 14.60% (which is desired in this case). For the RMSE parameter, the selection direction was desirable for all traits, with gains ranging from 0.19% (PIA) to 11.20% (FRY), while PIA showed a reduction of 4.39%. On the other hand, the MTMPS index showed potential to increase selection gains for the $S_{di}^{2}$ parameter in six traits, ranging from 3.44% (PH) to 19.40% (FRY). However, in the case of DMC, the MTMPS index did not result in an increase (-1.98%), indicating that the selection of genotypes did not yield desired gains for all traits. However, there was a reduction of -17.60% for PIA, which is desirable for mechanized planting.

**Table 5:** Selection differential of the selected genotypes for parameters $S_{di}^{2}$ (regression deviations) *R* ^2^ (regression determination coefficient) and RMSE (square root of the regression mean squared error) of the Eberhart and Russell model, 1966, with an economic weight of 65% for performance and 35% for stability, respectively.

Traits	$S_{di}^{2}$ /FA	Xo	Xs	GS	SG%	Direction	Target
Fresh root yield	FA1	23.80	28.60	4.62	19.40	increase	Yes
Dry root yield	FA1	7.49	8.71	1.18	15.80	increase	Yes
Shoot yield	FA1	20.20	22.10	1.82	8.99	increase	Yes
Harvest index	FA2	54.30	57.00	2.65	4.87	increase	Yes
Plant height	FA1	2.24	2.32	0.07	3.44	increase	Yes
Dry matter content	FA1	35.50	34.80	-0.70	-1.98	increase	No
Plant architecture	FA3	2.55	2.10	-0.45	-17.60	To decrease	Yes
	*R* ^2^ FA	Xo	Xs	GS	SG%	Direction	Target
Fresh root yield	FA1	23.80	28.40	4.51	18.90	increase	Yes
Dry root yield	FA1	7.49	8.75	1.22	16.20	increase	Yes
Shoot yield	FA2	20.20	23.10	2.78	13.80	increase	Yes
Harvest index	FA2	54.30	55.80	1.42	2.62	increase	Yes
Plant height	FA2	2.24	2.36	0.12	5.19	increase	Yes
Dry matter content	FA3	35.50	35.30	-0.22	-0.62	increase	No
Plant architecture	FA3	2.55	2.17	-0.37	-14.60	to decrease	Yes
	RMSE /FA	Xo	Xs	GS	SG%	Direction	Target
Fresh root yield	FA1	23.80	26.50	2.66	11.20	increase	Yes
Dry root yield	FA1	7.49	8.30	0.78	10.40	increase	Yes
Shoot yield	FA2	20.20	20.30	0.04	0.19	increase	Yes
Harvest index	FA2	54.30	56.90	2.51	4.62	increase	Yes
Plant height	FA2	2.24	2.36	0.11	5.15	increase	Yes
Dry matter content	FA3	35.50	35.70	0.16	0.46	increase	Yes
Plant architecture	FA3	2.55	2.44	-0.11	-4.39	to decrease	Yes

Xo , overall mean; Xs , mean of selected; GS , selection gain; SG% , selection gain in percentage considering multi-trait selection.

**Figure 5:**
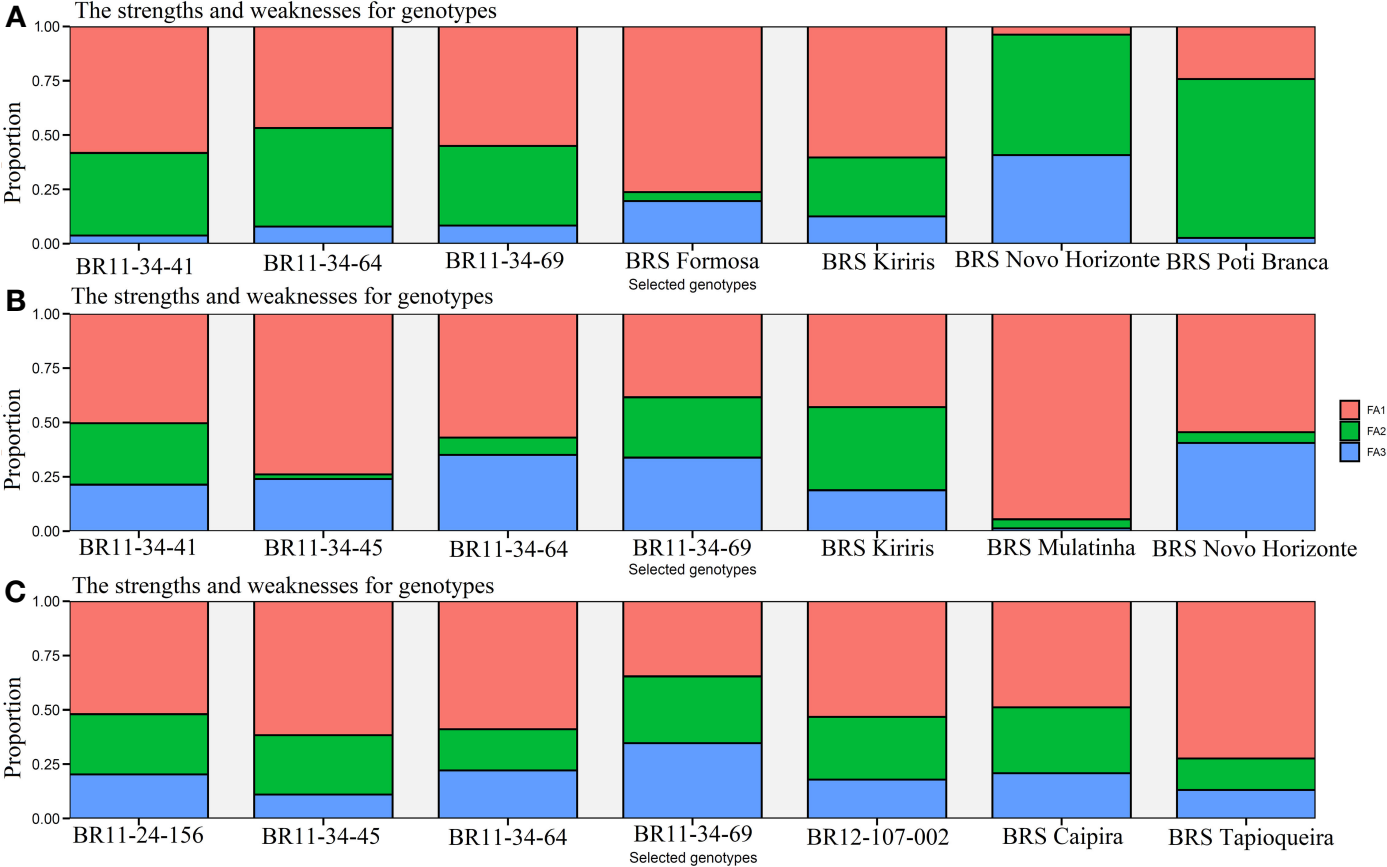
Cassava genotypes selected by the multi-trait index (MTMPS), based on the parameters: **(A)** $S_{di}^{2}$ (regression deviations), **(B)** *R* ^2^ (regression determination coefficient) and **(C)** for RMSE (square root of the mean squared error of the regression) of the model by Eberhart and Russell, 1966, with an economic weight of 65% for performance and 35% for stability respectively, considering a selection intensity of 30%, as well as the strengths and weaknesses of the genotypes taking into account the contribution proportion of each factor.

With a selection intensity of 30%, the MTMPS index enabled the selection of seven genotypes based on the $S_{di}^{2}$ , *R* ^2^, and RMSE parameters (**Figure 5**). The contribution of each factor determines its influence on the distance from the ideotype. Based on the $S_{di}^{2}\;$ parameter, the selected genotypes with the lowest MTMPS values are BRS Novo Horizonte (2.29), BRS Kiriris (3.14), BRS Formosa (3.14), BRS Poti Branca (3.25), BR11-34-69 (3.52), BR11-34-41 (3.61), and BR11-34-64 (3.67). FA2, which is more related to HI, contributes significantly to the distance of the BRS Novo Horizonte genotype from the ideotype (55.46%), while FA3, associated with PIA, contributes 40.78%. In contrast, genotypes BRS Formosa, BRS Kiriris, BR11-34-41, and BR11-34-69 have a higher contribution of ideotype distance in FA1 (associated with FRY, DRY, ShY, PH, and DMC), with FA1 contributions ranging from 54.97% to 76.28%. BRS Poti Branca has FA2 as the primary contributor (73.06%), while BR11-34-64 has a balanced contribution between FA1 and FA2 (46.76% and 45.31%, respectively).

For the *R* ^2^ parameter, the selected genotypes were BR11-34-69 (1.83), BRS Novo Horizonte (1.85), BRS Kiriris (2.15), BR11-34-45 (2.19), BR11-34-64 (2.45), BR11-34-41 (2.66), and BRS Mulatinha (2.85). The *R* ^2^ parameter highlights the selection of four genotypes in the final validation phase, with a significant contribution from FA1 (54.93%), primarily associated with FRY and DRY traits. Only BRS Novo Horizonte exhibits a balanced importance between FA1 and FA3, which is more related to DMC and PIA.

Regarding the RMSE parameter, the genotypes with lower MTMPS values were BR12-107-002 (2.88), BR11-24-156 (3.15), BR11-34-45 (2.71), BR11-34-69 (2.73), BRS Tapioqueira (3.26), BRS Caipira (3.09), and BR11-34-64 (3.10). In this case, there was a greater contribution of FA1 (54.52%) for the selected genotypes, which is associated with the productive attributes FRY and DRY. The contribution of FA1 ranged from 34.61% for BR11-34-69 to 72.37% for BRS Tapioqueira. FA2, which is associated with the traits ShY, HI, and PH, made an even contribution (25.48%) to all the genotypes selected by the RMSE parameter. Therefore, the MTMPS index proved to be highly useful for selecting and accurately classifying cassava genotypes based on stability and performance across multiple agronomic traits. It also identified the strengths and weaknesses of the selected genotypes through the contribution of each factor.

## 4 Discussion

### 4.1 GEI interaction and genetic parameters for agronomic traits in cassava

Significant progress has been made in cassava breeding programs, aiming to develop new varieties with improved genetic gains, performance, stability, and adaptability compared to traditional cultivars (Ceballos et al., 2012). GEI challenges efficient selection due to variable genotype rankings in different environments, especially for complex traits. This introduces bias and diminishes selection gains, necessitating multi-environment trials (Farshadfar et al., 2013).

The study underscores the challenge of selecting cassava genotypes that balance stability and high performance due to significant GEI effects. However, innovative approaches and understanding genetic parameters can help identify genotypes less influenced by environmental variations. The genotypic coefficient of variation ( CVɡ ) exceeded the residual coefficient of variation ( CVr ) for secondary traits and agronomic attributes like FRY and DMC, indicating favorable conditions for genotype selection in different breeding program phases. These findings align with previous research on FRY and DMC in various conditions (Adjebeng-Danquah et al., 2017).

Approximately 50% of the phenotypic variance in the studied traits is attributed to environmental effects and GEI interactions, reducing heritability. Secondary traits and DMC exhibit higher $H^{2}\;$ values (>0.40). While DMC is primarily influenced by additive effects (Wolfe et al., 2016), ShY and DRY are affected by non-additive genetic effects. Nonetheless, genetic gains can still be achieved through indirect selection, recurrent selection strategies, and exploiting heterotic effects.

The rɡe is relatively similar for FRY, ShY, DRY, and HI (≈0.54), while DMC, PH, and PIA show lower correlations (≈0.44). Heritability varies across traits, with most traits having relatively low estimates ranging from 0.30 (ShY) to 0.42 (PIA). DMC (0.57) and PH (0.50) exhibit higher heritability values. Secondary traits generally have higher heritability compared to primary traits, suggesting their potential for indirect selection of traits with greater agronomic importance.

### 4.2 Selection of genotypes via MPS

In the final validation phase, relying solely on production performance or stability information is not sufficient for efficient genotype recommendation, as it is possible to identify genotypes with low agronomic performance but high stability between environments, and vice versa (Yan and Tinker, 2006; Jiwuba et al., 2020). For example, some genotypes like BR11-24-156 and BRS Dourada showed yields below 20.0 t ha^-1^ of FRY and 6.79 t ha^-1^ of ShY but displayed high stability based on $S_{di}^{2}\;$ and $R^{2}$ parameters. Conversely, selecting genotypes with high performance does not necessarily guarantee high stability. Over 50% of evaluated genotypes fell into Group 3 (productive but unstable genotypes) for primary traits, including clones like BR11-34-41 (>31.0 t ha^-1^ FRY) and BR11-34-45 (>35.0% DMC), suggesting suitability for predictable environments.

The challenge of simultaneous selection for agronomic performance and yield stability has been reported by other authors in various crops using the WAASB model (weighted average of absolute scores) (Olivoto et al., 2019b; Nataraj et al., 2021; Sharifi et al., 2021). Specifically in cassava, Koundinya et al. (2021) encountered a high proportion of genotypes in both Group2 and Group3, highlighting the difficulty of simultaneous selection for performance and stability when using a multi-character index on 25 cassava genotypes across four growing conditions in India. Furthermore, a significant GEI effect was observed in the new genotypes for the four primary traits compared to the check varieties, as indicated by the $S_{di}^{2}\;$ parameter, where a higher proportion of checks were classified in Group1. Conversely, the stability parameters $R^{2}$ and RMSE grouped the new genotypes in Group1, indicating higher performance and stability. Weighting these parameters appropriately in VCU (variety value for cultivation and use) trials is essential for better genotype recommendations. Regarding secondary traits, except for HI, there was a greater grouping of new genotypes in Group 1 for traits like PH and PIA.

While yield stability index (YSI) and genotype stability index (GSI) have been proposed for selecting genotypes based on yield performance and stability, relying solely on the average stability value (ASV) parameter can lead to biased results. Additionally, these methods lack flexibility in economic weights or ranking scenarios. Additionally, these methods do not allow for varying economic weights or evaluating different ranking scenarios. The MPS selection index, on the other hand, leverages relationships between agronomic performance and production stability using conventional stability analysis methods. It aims to group genotypes with similar phenotypic behavior for both performance and stability. By utilizing the regression model proposed by Eberhart and Russell (1966) and applying economic weights ranging from 0 to 100, the MPS index identifies four clusters, representing genotypes with similar GEI patterns across evaluated environments for both primary and secondary selection traits.

The $S_{di}^{2}\;$ parameter corresponds to the regression variance, where $S_{di}^{2}\;$ = 0 indicates genotypes with high predictability (stability) and no GEI. The *R* ^2^ parameter represents the regression coefficient, with $R^{2}$ = 1 indicating genotypes with higher adaptability. The RMSE parameter is the root mean square of the regression error, where more stable genotypes have lower RMSE . These regression parameters effectively discriminate classification differences and facilitate the selection of genotypes with lower $S_{di}^{2}\;$ and RMSE and/or higher *R* ^2^, thereby achieving high productivity and stability. Conversely, genotypes that are more unstable and have low productivity can be identified based on their distance from the ideal values in the regression parameters. Analyzing the $S_{di}^{2}\;$ and $R^{2}$ parameters jointly can enhance interpretative efficiency, provide biological significance, explore GEI, and identify the optimal genotypes.

For primary traits, regardless of economic weight, BRS Novo Horizonte in Group 1 stood out due to its high agronomic performance and stability. Despite facing edaphoclimatic variations, it exhibited an average yield of 27.69 t ha^-1^, which is 80% higher than the average root yield in the state of Bahia (15.0 t ha^-1^ according to CONAB, 2020). It also showed DMC > 38% and PH > 2m, indicating high stability in production. Other genotypes in Group1, such as BR11-34-69 and BR11-34-41, demonstrated high stability based on the *R* ^2^ and RMSE parameters, along with FRY > 30 t ha^-1^ and DRY > 9 t ha^-1^, indicating consistent and productive performance. While BR11-24-156 displayed high predictability based on all three stability parameters, its agronomic performance values were moderate.

In Group1, the reference variety BRS Formosa exhibited high stability for CI (>62%), while the genotype BR12-107-002 showed stability for PIA (<2) based on all three stability parameters. On the other hand, genotypes in Group4 were characterized by unpredictability and low agronomic performance, suggesting the influence of GEI. For example, the genotype IAC-90 showed low yields (<16.0 t ha^-1^), ShY (9.0 t ha^-1^), and DMC (33%). BRS Verdinha exhibited a more branched growth (>4), which hinders mechanized planting, and a harvest index (HI) below 51%, along with average yield values. Overall, the MPS index represents a significant advancement over existing selection methods, as it identifies useful genetic relationships for breeders. It provides a robust methodological basis that is easy to manipulate and interpret for multi-character selection, with reduced sensitivity to outliers (Olivoto et al., 2017).

### 4.3 Selection of genotypes via multi-trait index - MTMPS

The use of truncated selection, which relies on information from a limited number of environments, has been commonly employed in cassava breeding programs (Ceballos et al., 2012). However, this approach has drawbacks, including potential loss of valuable alleles for other desirable traits (Cairns et al., 2013; Mohammadi et al., 2017). In recent years, as breeding programs have advanced and the production system has become more modernized, there has been an increasing demand for simultaneous improvement of multiple traits through multivariate approaches, driven by the evolving requirements of the cassava industry.

Recommendations based on multi-trait analysis are considered more reliable than single-trait analysis, particularly when the evaluated traits are highly correlated. For instance, a strong correlation was observed between FRY and DRY (>0.90) across all three stability parameters, consistent with existing literature (correlation of 0.97 - Diniz and Oliveira, 2019). There was also a significant correlation between FRY and ShY for the stability parameters $S_{di}^{2}\;$ and *R* ^2^, with correlation coefficients of r=0.70 and r=0.52, respectively. This corroborates reports by Ntawuruhunga and Dixon (2010) who found a correlation of 0.75 between FRY and ShY. Oliveira et al. (2017) reported positive and significant correlations of 0.70 between PH and YSI, and 0.71 between HI and YSI. They also noted negative correlations between FRY and YSI (-0.07) and between ShY and YSI (-0.23).

The yield components (FRY, ShY, DRY, and DMC) exhibit high genetic correlation and are strongly influenced by environmental variation (Ntawuruhunga and Dixon, 2010). Therefore, direct selection for these traits may not be advantageous due to their low heritability (average of 0.36 for the primary variables), which limits overall genetic gain. However, in cases where there are high magnitudes of correlation and heritability (average of 0.43 for secondary variables), it is possible that indirect measurement and selection based on initial characteristics of plant growth and development may be efficient in identifying desirable genotypes, thereby reducing the selection cycle.

Addressing multicollinearity issues is crucial to avoid bias in genetic parameter estimates during selection, especially when using conventional indices like Smith (1936). Therefore, it is crucial to extend conventional selection methods to encompass multiple traits in order to maximize genetic gains, taking into account the correlated genetic relationships among traits, as well as the presence of pleiotropy and genetic linkage (Malosetti et al., 2008). Additionally, assigning appropriate economic weights to agronomically important traits remains a challenge, often leading to suboptimal gains per generation (Cerón-Rojas et al., 2006; Olivoto et al., 2017). Establishing correlations between stability and agronomic performance for primary traits poses a significant challenge in cassava breeding.

The use of base and multiplicative selection indexes for multiple traits has been limited. The former does not incorporate genotypic and phenotypic variance-covariance matrices, thus ignoring GEI interactions, while the latter neglects economic weights. Furthermore, both base and multiplicative selection indexes fail to incorporate information on yield stability (Ntivuguruzwa et al., 2020). In contrast, the MTMPS index offers a multivariate approach using exploratory factor analysis to select genotypes based on the clustering of correlated traits, overcoming classic multicollinearity issues. It employs a more efficient linear mixed model (LMM) to predict genotypic responses, such as genetic parameters, thereby enhancing the reliability of clone grouping based on the index (Van Eeuwijk et al., 2016).

The MTMPS index allows genotype-ideotype selection (based on Euclidean distance) by considering multiple traits. The procedure involves the following steps: (i) using the correlation matrix between traits to identify eigenvalues (variance of principal components) and eigenvectors of genotypes (weights of traits explaining the variance determined by associated eigenvalues), (ii) performing principal component analysis on the characteristics to reduce the dimensionality of the data and identify the number of factors grouping the traits, and (iii) applying varimax rotation to estimate factorial loadings, considering eigenvalues >1, to interpret variable grouping and address multicollinearity concerns (Olivoto et al., 2017; Olivoto et al., 2019b). Additionally, the obtained communality values (representing the common variance explained by factors) ranged from 68.0% to 98.0% across all three parameters, indicating that the factors sufficiently represented an average of 88% of the total variation in the data.

The ideotype concept is crucial in breeding strategies, aiming to obtain genotypes with high performance and stability across multiple traits in a specific target environment (Yan, 2011). In cassava, the ideotype aims for maximum yield for primary and secondary traits, except for plant architecture, where smaller values are desirable. In this study, an economic weight representing 65% performance and 35% stability was used, employing the Eberhart and Russell (1966) regression model to compose the MTMPS index.

Using a selection intensity of 30%, the MTMPS index identified seven clones, most of which were new genotypes in the final validation phase. These selected clones demonstrated lower MTMPS values, indicating their proximity to the ideotype. Genotypes such as BR11-34-69 and BR11-34-64 were chosen based on all three stability parameters and were deemed suitable for cultivation in diverse environments. On the other hand, genotypes BRS Novo Horizonte, BRS Kiriris, and BR11-34-41 were selected based on the $S_{di}^{2}\;$ and *R* ^2^ parameters, respectively, as they exhibited low or zero GEI interaction even in the face of environmental variations.

Analyzing strengths and weaknesses revealed that yield components had a greater contribution, followed by secondary variables, in explaining and influencing genotype selection. The traits DRY (0.96), ShY (0.48), and HI (0.38), which represent yield components, strongly influenced the highest FRY (*R* ^2^ >0.97). FRY and DRY were the most significant contributors to the first factor (FA1) of the MTMPS index in all three stability parameters, accounting for more than 56% of the contribution. Thus, the selection process prioritized cassava genotypes with higher potential for acceptance in starch and flour processing agro-industries, which desire higher FRY and DRY values. Notable improved genotypes included BRS Novo Horizonte, BRS Tapioqueira, and two genotypes in the final validation phase, BR11-34-69 and BR11-34-64.

In summary, both MPS and MTMPS techniques proved to be effective in exploring GEI in cassava breeding, leading to improved performance and productive stability across all primary and secondary traits considered in the multi-trait evaluation.

### 4.4 Future perspectives

The MPS and MTMPS indices provide valuable insights into the optimization potential of the cassava breeding program, aiding in the identification and selection of genotypes at different stages of the program. The next steps involve incorporating new trials conducted in diverse growing regions to assess the accuracy of the MPS and MTMPS indices in the presence of increased GEI. Additionally, different stability models will be explored, considering various scenarios of economic weights. This analysis aims to identify genotypes within the evaluated set (including intermediate and final selection stages, as well as improved and unimproved varieties) that exhibit potential for simultaneous genetic gains in both quantitative traits (such as germination vigor, soil coverage, tolerance to herbicides, root and aboveground productivity, starch content) and qualitative traits (such as carotenoid content, root dry matter, cooking ability) of agronomic interest for both industry and fresh consumption. By combining these approaches, the time required for recommending new genotypes can be reduced, while generating valuable information about parents with contrasting traits, stability in productive attributes, and a higher likelihood of expressing heterosis in the offspring.

## 5 Conclusion

Utilizing multivariate selection yielded significant insights into estimating genetic parameters, resulting in an average heritability ( $H^{2}$ ) of 0.39, and effectively gauging the extent of genotype-by-environment interaction ( $\sigma_{ɡxe}^{2}$ ) across diverse traits. The MPS index, utilizing varying weights (ranging from 0 to 100) assigned through the Eberhart and Russell (1966) regression model, efficiently and robustly explored the GEI in the multi-environment trial (MET) data. This method adeptly formed clusters of genotypes that exhibited heightened congruence in both performance metrics and production stability.

Group 1 consisted of highly productive and stable genotypes for primary traits like FRY, including varieties such as BRS Novo Horizonte and BR11-34-69, as well as for DMC with genotypes BRS Novo Horizonte and BR12-107-002. Regarding secondary characteristics like HI and PIA, genotypes BRS Formosa and BR12-107-002 showed the highest stability and better agronomic performance. Therefore, employing methods that consider both stability and productive performance can enhance the reliability of recommending new cassava cultivars. Moreover, using the MTMPS index with a selection intensity of 30% led to the identification of seven genotypes with higher stability based on the parameters of Eberhart and Russell (1966). Among these, four were novel cultivars, chosen via *R* ^2^ and RMSE metrics across multiple traits. Overall, the application of multivariate selection, in conjunction with the MPS and MTMPS indices, effectively demonstrated its prowess in estimating genetic parameters, capturing the nuances of genotype-by-environment interaction, and successfully pinpointing genotypes showcasing amplified stability and performance within cassava breeding initiatives.

## Data availability statement

The original contributions presented in the study are included in the article/**Supplementary Material**. Further inquiries can be directed to the corresponding author.

## Author contributions

JS: Conceptualization, Data curation, Formal Analysis, Methodology, Writing – original draft. TO: Formal Analysis, Writing – review & editing. MC: Writing – review & editing, Data curation, Validation. EdO: Writing – review & editing, Conceptualization, Funding acquisition, Methodology, Project administration, Supervision.

## Supplementary material

Supplementary Figure 1 Means of cassava clones in each of the 47 field trials evaluated for the following traits: **(A)** fresh root yield (FRY, t ha^-1^), **(B)** shoot yield (ShY, t ha^-1^), **(C)** dry root yield (DRY, t ha^-1^), **(D)** root dry matter content (DMC, %), **(E)** Harvest index (HI, %), **(F)**, Plant height (PH, m), **(G)** plant size (PIA, scale 1 to 5), and cassava evaluated with 22 genotypes in 47 environments.

Supplementary Figure 2 Mean and phenotypic variation of individual environments for fresh root yield (FRY, t ha^-1^), shoot yield (ShY, t ha^-1^), dry root yield (DRY, t ha^-1^), root dry matter content (DMC, %), plant architecture (PIA, scale 1 to 5), harvest index (HI, %) and plant height (PH, m) in 22 cassava genotypes evaluated in 47 environments.

Supplementary Figure 3 Pearson’s correlation between the agronomic variables for the mean performance and stability (MPS) index for the parameters: **(A)** $S_{di}^{2}$ (regression deviations), **(B)** *R* ^2^ (regression determination coefficient) and **(C)** RMSE (square root of the regression mean square error) of the model by Eberhart and Russell, 1966, with an economic weight of 65% for performance and 35% for stability, respectively.
