# Supplementary figures and images for: Multi-trait selection in multi-environments for performance and stability in cassava genotypes
Source: Front Plant Sci. 2023 Oct 30;14:1282221. doi: 10.3389/fpls.2023.1282221 (PMC10642803; doi:10.3389/fpls.2023.1282221)

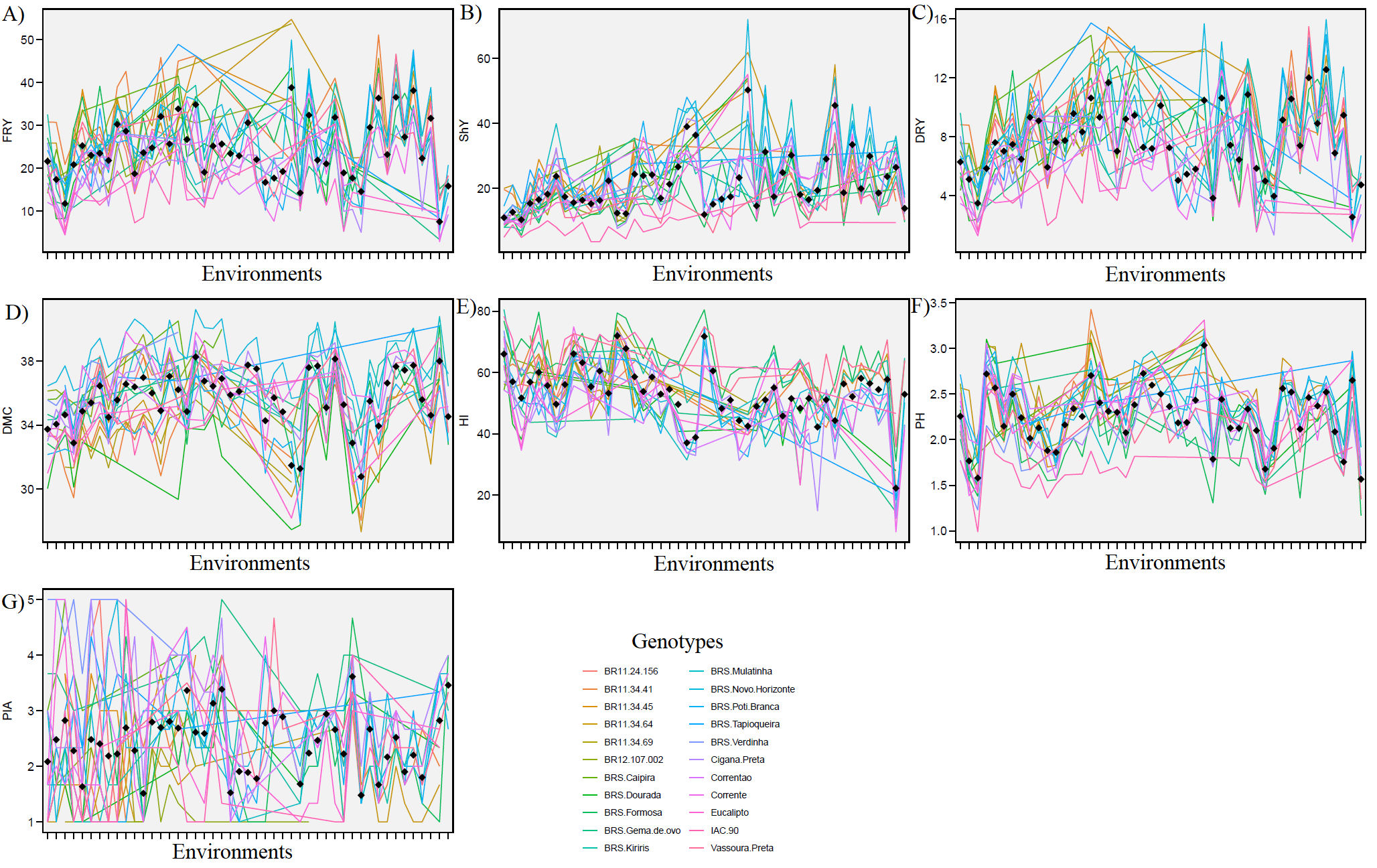

Supplement: Supplementary Figure 1 — Means of cassava clones in each of the 47 field trials evaluated for the following traits: (A) fresh root yield (FRY, t ha-1), (B) shoot yield (ShY, t ha-1), (C) dry root yield (DRY, t ha-1), (D) root dry matter content (DMC, %), (E) Harvest index (HI, %), (F), Plant height (PH, m), (G) plant size (PIA, scale 1 to 5), and cassava evaluated with 22 genotypes in 47 environments. [file Image_1.tif]

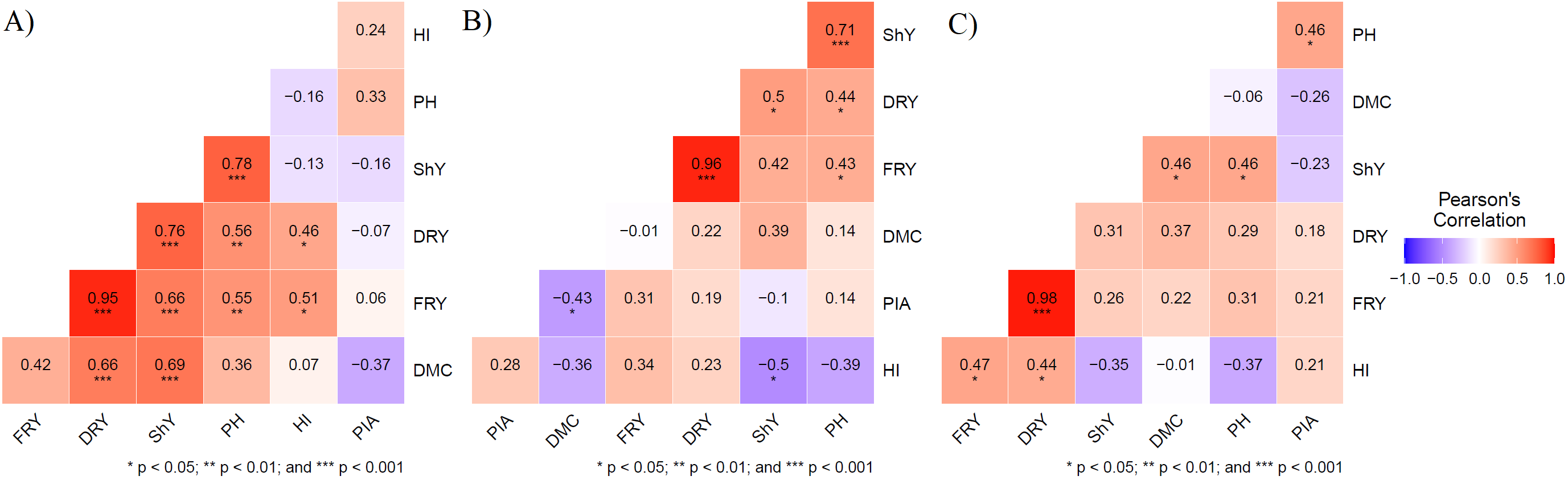

Supplement: Supplementary Figure 3 — Pearson’s correlation between the agronomic variables for the mean performance and stability (MPS) index for the parameters: (A) Sdi2 (regression deviations), (B) R 2 (regression determination coefficient) and (C) RMSE (square root of the regression mean square error) of the model by Eberhart and Russell, 1966, with an economic weight of 65% for performance and 35% for stability, respectively. [file Image_3.tif]
