# Supplementary material for: Multi-trait selection in multi-environments for performance and stability in cassava genotypes
Source: Front Plant Sci. 2023 Oct 30;14:1282221. doi: 10.3389/fpls.2023.1282221 (PMC10642803; doi:10.3389/fpls.2023.1282221)
Supplement: Supplementary file 4 [file Table_1.docx]

**Table S1.** List of the cassava field trials evaluated from 2016 to 2021, for the four agronomic traits.

| order | Trial | Year | Type of trial | Location | State | Wind/speed (ms^-1^) | Solar/rad (MJ/m^-2^ day^-1^) | Altitude (m) | Latitude | Longitude | Type soil |
| --- | --- | --- | --- | --- | --- | --- | --- | --- | --- | --- | --- |
| 1 | 2016.EA.NH | 2016 | Advanced yield trial | Laje-Novo Horizonte | Bahia | 1.89 | 18.51 | 190 | 13°08′36″ | 39°25′46″ | LYRd |
| 2 | 2016.EA.SA | 2016 | Advanced yield trial | Santo Amaro | Bahia | 2.38 | 19.12 | 42 | 12°32′48″ | 38°42′43″ | V |
| 3 | 2016.ERU.SA | 2016 | Uniform Yield Test | Santo Amaro | Bahia | 2.38 | 19.12 | 42 | 12°32′48″ | 38°42′43″ | V |
| 4 | 2017.ERU.NH | 2017 | Uniform Yield Test | Laje-Novo Horizonte | Bahia | 1.99 | 18.67 | 190 | 13°08′36″ | 39°25′46″ | LYRd |
| 5 | 2017.ERU.SA | 2017 | Uniform Yield Test | Santo Amaro | Bahia | 2.43 | 19.26 | 42 | 12°32′48″ | 38°42′43″ | V |
| 6 | 2017.ERU.SJ | 2017 | Uniform Yield Test | Laje São Jorge | Bahia | 1.99 | 18.67 | 190 | 13°08′36″ | 39°25′46″ | LYRd |
| 7 | 2017.ERU.SV | 2017 | Uniform Yield Test | Laje Sombra Verde | Bahia | 1.99 | 18.67 | 190 | 13°08′36″ | 39°25′46″ | LYRd |
| 8 | 2018.ERU.RA1 | 2018 | Uniform Yield Test | Laje Rio de areia 1 | Bahia | 187 | 17.98 | 190 | 13°08′47″ | 39°17′58″ | LYRd |
| 9 | 2018.ERU.RA2 | 2018 | Uniform Yield Test | Laje Rio de areia 2 | Bahia | 187 | 17.98 | 190 | 13°08′47″ | 39°17′58″ | LYRd |
| 10 | 2018.ERU.PP | 2018 | Uniform Yield Test | Laje Propriedade particular | Bahia | 187 | 17.98 | 190 | 13°08′47″ | 39°17′58″ | LYRd |
| 11 | 2018.ERU.UFRB | 2018 | Uniform Yield Test | Cruz das Almas-UFRB | Bahia | 2.72 | 18.38 | 220 | 12°39′11″ | 39°07′19″ | LYRd |
| 12 | 2019.ERU.GA | 2019 | Uniform Yield Test | Laje Gavião | Bahia | 1.89 | 19.38 | 190 | 13°09′52″ | 39°25′59″ | LYRd |
| 13 | 2019.ERU.NH | 2019 | Uniform Yield Test | Laje-Novo Horizonte | Bahia | 1.89 | 19.38 | 190 | 13°09′52″ | 39°25′59″ | LYRd |
| 14 | 2019.ERU.NR | 2019 | Uniform Yield Test | Valença | Bahia | 1.89 | 19.38 | 39 | 13°22′26″ | 39°04′30″ | LAd |
| 15 | 2019.ERU.RA1 | 2019 | Uniform Yield Test | Laje Rio de areia 1 | Bahia | 1.89 | 19.38 | 190 | 13°09′52″ | 39°25′59″ | LYRd |
| 16 | 2019.ERU.RA2 | 2019 | Uniform Yield Test | Laje Rio de areia 2 | Bahia | 1.89 | 19.38 | 190 | 13°09′52″ | 39°25′59″ | LYRd |
| 17 | 2019.ERU.M.RA2 | 2019 | Uniform Yield Test | Laje Rio de areia 2 | Bahia | 1.89 | 19.38 | 190 | 13°09′52″ | 39°25′59″ | LYRd |
| 18 | 2019.ERU.UFRB | 2019 | Uniform Yield Test | Cruz das Almas-UFRB | Bahia | 2.71 | 18.98 | 220 | 12°39′11″ | 39°07′19″ | LYRd |
| 19 | 2019.ERU.M.UFRB | 2019 | Uniform Yield Test | Cruz das Almas-UFRB | Bahia | 2.71 | 18.98 | 220 | 12°39′11″ | 39°07′19″ | LYRd |
| 20 | 2020.EA.UFRB | 2020 | Advanced yield trial | Cruz das Almas-UFRB | Bahia | 2.61 | 17.32 | 220 | 12°39′11″ | 39°07′19″ | LYRd |
| 21 | 2020.EA.RA1 | 2020 | Advanced yield trial | Laje Rio de areia 1 | Bahia | 1.87 | 17.62 | 190 | 13°39′52″ | 39°25′59″ | LYRd |
| 22 | 2020.ERU.UFV | 2020 | Uniform Yield Test | Florestal-UFV | Minas Gerais | 2.42 | 20.6 | 815 | 19°53'12'' | 44°25'56″ | AYRD |
| 23 | 2020.ERU.GM | 2020 | Uniform Yield Test | Governador Mangabeira | Bahia | 2.61 | 17.32 | 200 | 12°34′23″ | 38°42′53″ | LYRd |
| 24 | 2020.ERU.NH2A | 2020 | Uniform Yield Test | Laje-Novo Horizonte-2A | Bahia | 1.87 | 17.62 | 190 | 13°39′52″ | 39°25′59″ | LYRd |
| 25 | 2020.ERU.NH2B | 2020 | Uniform Yield Test | Laje-Novo Horizonte-2B | Bahia | 1.87 | 17.62 | 190 | 13°39′52″ | 39°25′59″ | LYRd |
| 26 | 2020.ERU.UFRB | 2020 | Uniform Yield Test | Cruz das Almas-UFRB | Bahia | 2.61 | 17.32 | 220 | 12°39′11″ | 39°07′19″ | LYRd |
| 27 | 2020.ERU.RA1 | 2020 | Uniform Yield Test | Laje Rio de areia 1 | Bahia | 1.87 | 17.62 | 190 | 13°39′52″ | 39°25′59″ | LYRd |
| 28 | 2021.EA.UFRB | 2021 | Advanced yield trial | Cruz das Almas-UFRB | Bahia | 2.29 | 15.82 | 220 | 12°39′11″ | 39°07′19″ | LYRd |
| 29 | 2021.EA.NH | 2021 | Advanced yield trial | Laje-Novo Horizonte | Bahia | 2.18 | 17.17 | 190 | 13°09′52″ | 39°25′59″ | LYRd |
| 30 | 2021.EA.RA1 | 2021 | Advanced yield trial | Laje Rio de areia 1 | Bahia | 2.18 | 17.17 | 190 | 13°09′52″ | 39°25′59″ | LYRd |
| 31 | 2021.EA.GS.AL | 2021 | Advanced yield trial | Alagoinhas | Bahia | 2.29 | 18.93 | 230 | 12° 07' 13'' | 38°24'35'' | PYR |
| 32 | 2021.EA.GS.NH | 2021 | Advanced yield trial | Laje-Novo Horizonte | Bahia | 2.18 | 17.17 | 190 | 13°09′52″ | 39°25′59″ | LYRd |
| 33 | 2021.EA.GS.UFRB | 2021 | Advanced yield trial | Cruz das Almas-UFRB | Bahia | 2.29 | 15.82 | 220 | 12°39′11″ | 39°07′19″ | LYRd |
| 34 | 2021.EA.GS.RA1 | 2021 | Advanced yield trial | Laje Rio de areia 1 | Bahia | 2.18 | 17.17 | 190 | 13°09′52″ | 39°25′59″ | LYRd |
| 35 | 2021.ERU.UFRB | 2021 | Uniform Yield Test | Cruz das Almas-UFRB | Bahia | 2.29 | 15.82 | 220 | 12°39′11″ | 39°07′19″ | LYRd |
| 36 | 2021.ERU.RA1 | 2021 | Uniform Yield Test | Laje Rio de areia 1 | Bahia | 2.18 | 17.17 | 190 | 13°09′52″ | 39°25′59″ | LYRd |
| 37 | 2021.ERU.NH1 | 2021 | Uniform Yield Test | Laje-Novo Horizonte1 | Bahia | 2.18 | 17.17 | 190 | 13°09′52″ | 39°25′59″ | LYRd |
| 38 | 2021.ERU.NH2 | 2021 | Uniform Yield Test | Laje-Novo Horizonte2 | Bahia | 2.18 | 17.17 | 190 | 13°09′52″ | 39°25′59″ | LYRd |
| 39 | 2021.ERU.NH3 | 2021 | Uniform Yield Test | Laje-Novo Horizonte3 | Bahia | 2.18 | 17.17 | 190 | 13°09′52″ | 39°25′59″ | LYRd |
| 40 | 2021.ERU.NH4 | 2021 | Uniform Yield Test | Laje-Novo Horizonte4 | Bahia | 2.18 | 17.17 | 190 | 13°09′52″ | 39°25′59″ | LYRd |
| 41 | 2021.ERU.AL | 2021 | Uniform Yield Test | Alagoinhas | Bahia | 2.29 | 18.93 | 230 | 12° 07' 13'' | 38°24'35'' | PYR |
| 42 | 2021.ERU.RIOS | 2021 | Uniform Yield Test | Entre Rios | Bahia | 1.1 | 16.66 | 162 | 11º56'31" | 38º05'04" | PYR |
| 43 | 2021.ERU.ALC | 2021 | Uniform Yield Test | Alcobaça | Bahia | 4.63 | 16.95 | 16 | 17°31'21'' | 39°11'53'' | PDQS |
| 44 | 2021.ERU.ITAM | 2021 | Uniform Yield Test | Itamarajú | Bahia | 1.00 | 18.13 | 112 | 17°02'21'' | 39°31'52'' | LYRd |
| 45 | 2021.ERU.NH1 | 2021 | Uniform Yield Test | Laje-Novo Horizonte1 | Bahia | 2.18 | 17.17 | 190 | 13°09′52″ | 39°25′59″ | LYRd |
| 46 | 2021.ERU.NH2 | 2021 | Uniform Yield Test | Laje-Novo Horizonte2 | Bahia | 2.18 | 17.17 | 190 | 13°09′52″ | 39°25′59″ | LYRd |
| 47 | 2021.ERU.UFGD | 2021 | Uniform Yield Test | Dourados-UFGD | Mato Grosso do Sul | 0.24 | 18.17 | 469 | 22º11'16" | 54º54'20" | LRDF |
