# Supplementary material for: Multi-trait selection in multi-environments for performance and stability in cassava genotypes
Source: Front Plant Sci. 2023 Oct 30;14:1282221. doi: 10.3389/fpls.2023.1282221 (PMC10642803; doi:10.3389/fpls.2023.1282221)
Supplement: Supplementary file 5 [file Table_2.docx]

**Table S2.** Summary of phenotypic means ($\bar{X}$) and the best linear unbiased predictor (Blupg) of the 22 cassava genotypes evaluated for seven agronomics traits in 47 environments.

| Genotypes | FRY | | ShY | | DRY | | DMC | | PIA | | HI | | PH | |
| --- | --- | --- | --- | --- | --- | --- | --- | --- | --- | --- | --- | --- | --- | --- |
|  | $\bar{X}$ | Blupg | $\bar{X}$ | Blupg | $\bar{X}$ | Blupg | $\bar{X}$ | Blupg | $\bar{X}$ | Blupg | $\bar{X}$ | Blupg | $\bar{X}$ | Blupg |
| BR11-34-69 | 32.4 | 6.5 | 19.4 | 0.4 | 9.3 | 1.3 | 33.2 | -2.3 | 1.7 | -0.8 | 63.2 | 5.3 | 2.5 | 0.2 |
| BR11-34-41 | 31.1 | 6.0 | 22.6 | 1.5 | 9.0 | 1.2 | 33.5 | -2.1 | 2.0 | -0.5 | 57.8 | 3.2 | 2.3 | 0.1 |
| BRS Formosa | 28.4 | 4.3 | 16.4 | -3.4 | 8.9 | 1.3 | 35.4 | -0.3 | 2.3 | -0.3 | 64.1 | 9.3 | 2.0 | -0.2 |
| BRS Kiriris | 28.3 | 3.8 | 19.5 | -3.3 | 8.9 | 1.2 | 35.4 | 0.0 | 2.2 | -0.3 | 59.8 | 7.6 | 2.3 | 0.0 |
| BR11-34-64 | 28.4 | 3.5 | 23.2 | 2.1 | 8.3 | 0.5 | 33.8 | -1.8 | 1.6 | -0.9 | 55.4 | 1.0 | 2.5 | 0.3 |
| BRS Novo Horizonte | 27.4 | 3.4 | 27.7 | 6.3 | 9.4 | 1.8 | 38.2 | 2.6 | 2.7 | 0.2 | 50.7 | -2.7 | 2.3 | 0.1 |
| BR11-34-45 | 28.4 | 2.9 | 23.4 | 4.0 | 8.8 | 0.9 | 35.9 | 0.3 | 2.8 | 0.3 | 55.3 | -2.1 | 2.3 | 0.0 |
| BRS Caipira | 25.8 | 2.3 | 20.7 | 2.1 | 8.9 | 1.2 | 37.3 | 2.0 | 3.7 | 1.0 | 55.8 | -0.4 | 2.2 | -0.2 |
| BRS Tapioqueira | 23.7 | 1.9 | 20.8 | 1.8 | 7.7 | 0.8 | 36.2 | 0.8 | 3.3 | 0.8 | 51.7 | -0.8 | 2.4 | 0.1 |
| Vassoura Preta | 25.1 | 1.3 | 16.0 | -5.0 | 8.1 | 0.5 | 36.0 | 0.4 | 2.5 | 0.1 | 61.4 | 8.4 | 2.1 | -0.2 |
| BRS Poti Branca | 24.0 | 0.3 | 25.7 | 4.5 | 7.3 | -0.1 | 34.4 | -1.2 | 2.2 | -0.3 | 48.2 | -5.1 | 2.4 | 0.2 |
| BR11-24-156 | 22.9 | -0.3 | 16.8 | -0.6 | 7.2 | 0.0 | 36.4 | 0.8 | 2.7 | 0.3 | 57.5 | 0.7 | 2.3 | 0.1 |
| BRS Mulatinha | 23.1 | -0.7 | 25.7 | 4.4 | 7.7 | 0.2 | 37.3 | 1.6 | 2.2 | -0.3 | 48.3 | -4.7 | 2.4 | 0.2 |
| BRS Verdinha | 21.1 | -1.3 | 15.7 | -0.7 | 6.8 | -0.1 | 36.6 | 1.6 | 4.7 | 2.2 | 57.2 | -0.2 | 1.8 | -0.4 |
| BRS Dourada | 21.3 | -1.7 | 22.7 | 0.9 | 6.2 | -1.1 | 32.4 | -2.8 | 2.4 | -0.3 | 47.6 | -4.1 | 2.4 | 0.1 |
| Corrente | 21.9 | -2.0 | 21.3 | 0.5 | 7.2 | -0.4 | 36.7 | 0.9 | 2.8 | 0.2 | 50.8 | -3.2 | 2.3 | 0.1 |
| Correntão | 20.3 | -2.5 | 23.4 | 2.5 | 6.6 | -0.7 | 35.7 | 0.3 | 2.4 | -0.1 | 46.4 | -6.8 | 2.4 | 0.2 |
| BR12-107-002 | 24.1 | -2.8 | 17.4 | -3.3 | 7.9 | -0.6 | 37.2 | 1.3 | 1.3 | -1.2 | 59.3 | 1.9 | 2.3 | 0.1 |
| Cigana Preta | 19.0 | -4.3 | 21.1 | 0.7 | 6.2 | -1.2 | 36.1 | 0.5 | 3.0 | 0.4 | 47.6 | -6.1 | 2.1 | -0.1 |
| BRS Gema de ovo | 16.9 | -5.7 | 20.0 | -1.1 | 5.6 | -1.7 | 36.1 | 0.4 | 3.7 | 0.8 | 44.7 | -7.0 | 2.1 | -0.2 |
| IAC-90 | 15.2 | -6.9 | 8.0 | -9.9 | 4.7 | -2.5 | 34.4 | -1.4 | 1.9 | -0.7 | 65.3 | 10.0 | 1.6 | -0.5 |
| Eucalipto | 14.8 | -7.8 | 17.2 | -4.3 | 4.4 | -2.7 | 33.7 | -1.6 | 2.1 | -0.6 | 47.2 | -4.4 | 2.3 | 0.0 |

FRY: fresh root yield (t ha^-1^), ShY: shoot yield (t ha^-1^), DRY: dry root yield (t ha^-1^), DMC: root dry matter content (%), HI: harvest index (%), PH: plant height (m), PIA: plant size (scale 1 to 5).
