# Supplementary material for: Multi-trait selection in multi-environments for performance and stability in cassava genotypes
Source: Front Plant Sci. 2023 Oct 30;14:1282221. doi: 10.3389/fpls.2023.1282221 (PMC10642803; doi:10.3389/fpls.2023.1282221)
Supplement: Supplementary file 6 [file Table_3.docx]

**Table S3**. Pearson's correlation between the agronomic traits for the mean performance and stability (MPS) index for the parameters $S_{di}^{2}$ (regression deviations), B) $R^{2}$ (regression determination coefficient) and C) $RMSE$ (regression mean square error square root) from the model by Eberhart and Russell, 1966, with an economic weight of 65% for performance and 35% for stability respectively, and path analysis, values with significance highlighted in bold.

| $S_{di}^{2}$ | FRY | ShY | HI | PH | DMC | DRY |  |  |
| --- | --- | --- | --- | --- | --- | --- | --- | --- |
| Shoot yield | **0.66^***^** |  |  |  |  |  |  |  |
| Harvest index | **0.51^*^** | -0.13 |  |  |  |  |  |  |
| Plant height | **0.55^**^** | **0.78^***^** | -0.16 |  |  |  |  |  |
| Dry matter content | 0.42 | **0.69^***^** | 0.07 | 0.36 |  |  |  |  |
| Dry root yield | **0.95^***^** | **0.76^***^** | **0.46^*^** | **0.56^**^** | **0.66^***^** |  |  |  |
| Plant architecture | 0.06 | -0.16 | 0.24 | 0.33 | -0.37 | -0.07 |  |  |
| $R^{2}$ | FRY | ShY | HI | PH | DMC | DRY |  |  |
| Shoot yield | 0.42 |  |  |  |  |  |  |  |
| Harvest index | 0.34 | **-0.50^*^** |  |  |  |  |  |  |
| Plant height | **0.43^*^** | **0.71^***^** | -0.39 |  |  |  |  |  |
| Dry matter content | 0.01 | 0.39 | -0.36 | 0.14 |  |  |  |  |
| Dry root yield | **0.96^***^** | **0.50^*^** | 0.26 | **0.44^*^** | 0.22 |  |  |  |
| Plant architecture | 0.31 | -0.01 | 0.28 | 0.28 | **-0.43^*^** | 0.19 |  |  |
| $RMSE$ | FRY | ShY | HI | PH | DMC | DRY |  |  |
| Shoot yield | 0.26 |  |  |  |  |  |  |  |
| Harvest index | **0.47^*^** | -0.35 |  |  |  |  |  |  |
| Plant height | 0.31 | **0.46^*^** | -0.37 |  |  |  |  |  |
| Dry matter content | 0.22 | 0.46^*^ | -0.01 | -0.06 |  |  |  |  |
| Dry root yield | **0.98^***^** | 0.31 | **0.44^*^** | 0.29 | 0.37 |  |  |  |
| Plant architecture | 0.21 | -0.23 | 0.21 | **0.46^*^** | -0.26 | 0.18 |  |  |
|  | Path analysis | | | | | | | |
|  | ShY | HI | PH | DMC | DRY | PIA | linear | VIF |
| Shoot yield | 0.1450 | -0.0766 | 0.0091 | -0.0203 | 0.4405 | -0.0004 | **0.5073** | **7.31** |
| Harvest index | -0.0760 | 0.1462 | -0.0043 | 0.0059 | 0.3340 | 0.0021 | 0.4078 | 6.65 |
| Plant height | 0.0804 | -0.0383 | 0.0163 | -0.0118 | 0.2962 | 0.0030 | 0.3459 | 1.58 |
| Dry matter content | 0.0168 | -0.0049 | 0.0011 | -0.1750 | 0.2816 | -0.0022 | 0.1174 | 1.40 |
| Dry root yield | 0.0715 | 0.0547 | 0.0054 | -0.0552 | 0.8928 | 0.0003 | **0.9695** | **6.91** |
| Plant architecture | 0.0039 | -0.0205 | -0.0034 | -0.0265 | -0.0183 | -0.0147 | -0.0795 | 1.15 |

*p< 0.05, ** p<0.01 e ***p<0.001
