# Supplementary material for: Multi-trait selection in multi-environments for performance and stability in cassava genotypes
Source: Front Plant Sci. 2023 Oct 30;14:1282221. doi: 10.3389/fpls.2023.1282221 (PMC10642803; doi:10.3389/fpls.2023.1282221)
Supplement: Supplementary file 7 [file Table_4.docx]

**Table S4**. Scores of the 22 genotypes and for the ideotype (ID) and the relative contribution of the MTMPS index to the genotypes for the three factors (selected genotypes are in bold)

| Genotypes | Factor scores $S_{di}^{2}$ | | | Contribution factor | | | Index |  | Factor scores $R^{2}$ | | | Contribution factor | | | Index |  | Factor scores $RMSE$ | | | Contribution factor | | | Index |
| --- | --- | --- | --- | --- | --- | --- | --- | --- | --- | --- | --- | --- | --- | --- | --- | --- | --- | --- | --- | --- | --- | --- | --- |
|  | FA1 | FA2 | FA3 | FA1 | FA2 | FA3 | MTMPS |  | FA1 | FA2 | FA3 | FA1 | FA2 | FA3 | MTMPS |  | FA1 | FA2 | FA3 | FA1 | FA2 | FA3 | MTMPS |
| BR11-34-69 | **-3,5** | **-3,0** | **3,8** | **55,0** | **36,7** | **8,3** | **3,5** |  | **-5,4** | **1,6** | **1,6** | **38,4** | **27,8** | **33,8** | **1,8** |  | **-5,7** | **-3,1** | **2,4** | **34,6** | **30,7** | **34,7** | **2,7** |
| BRS Novo Horizonte | **-6,2** | **-3,1** | **2,0** | **3,8** | **55,5** | **40,8** | **2,3** |  | **-5,2** | **2,6** | **-0,5** | **54,5** | **5,0** | **40,5** | **1,9** |  | -4,5 | -3,8 | -0,8 | 55,4 | 14,2 | 30,4 | 3,3 |
| BRS Kiriris | **-3,5** | **-3,7** | **2,7** | **60,3** | **27,1** | **12,5** | **3,1** |  | **-5,1** | **1,1** | **1,2** | **43,0** | **38,2** | **18,8** | **2,2** |  | -4,6 | -2,4 | 1,4 | 49,9 | 38,4 | 11,8 | 3,5 |
| BR11-34-45 | -3,6 | -2,1 | 2,0 | 39,9 | 41,2 | 18,9 | 4,2 |  | **-4,5** | **2,4** | **-0,1** | **74,0** | **2,1** | **23,9** | **2,2** |  | **-4,9** | **-3,4** | **0,4** | **61,7** | **27,3** | **11,0** | **2,7** |
| BR11-34-64 | **-3,7** | **-2,4** | **3,8** | **46,8** | **45,3** | **7,9** | **3,7** |  | **-4,6** | **2,2** | **1,9** | **57,0** | **7,9** | **35,1** | **2,5** |  | **-4,6** | **-3,6** | **1,8** | **59,0** | **19,0** | **22,1** | **3,1** |
| BR11-34-41 | **-3,3** | **-3,0** | **3,1** | **58,3** | **38,0** | **3,7** | **3,6** |  | **-4,5** | **1,3** | **1,5** | **50,4** | **28,3** | **21,4** | **2,7** |  | -4,4 | -2,5 | 1,4 | 53,3 | 36,1 | 10,6 | 3,6 |
| BRS Mulatinha | -4,0 | -1,4 | 2,4 | 34,5 | 52,3 | 13,3 | 4,3 |  | **-3,8** | **2,6** | **0,5** | **94,6** | **4,1** | **1,3** | **2,9** |  | -3,7 | -3,3 | -0,2 | 62,4 | 20,8 | 16,8 | 4,0 |
| BRS Tapioqueira | -4,0 | -2,5 | 1,7 | 36,3 | 37,9 | 25,8 | 3,7 |  | -4,4 | 1,3 | -0,9 | 45,2 | 24,9 | 29,9 | 2,9 |  | **-4,2** | **-3,9** | **0,2** | **72,4** | **14,5** | **13,1** | **3,3** |
| BRS Caipira | -3,7 | -2,7 | 0,9 | 36,1 | 30,7 | 33,3 | 4,3 |  | -4,4 | 1,9 | -1,3 | 47,7 | 12,2 | 40,1 | 2,9 |  | **-4,9** | **-3,0** | **-0,3** | **48,9** | **30,4** | **20,8** | **3,1** |
| BR12-107-002 | -3,1 | -2,2 | 3,3 | 54,1 | 45,7 | 0,3 | 4,2 |  | -3,8 | 1,7 | 0,6 | 76,3 | 22,8 | 0,9 | 2,9 |  | **-4,9** | **-3,2** | **1,6** | **53,2** | **28,9** | **17,9** | **2,9** |
| BRS Poti Branca | **-5,3** | **-1,9** | **3,2** | **24,2** | **73,1** | **2,7** | **3,3** |  | -3,7 | 2,5 | 1,1 | 83,6 | 0,5 | 16,0 | 3,0 |  | -3,2 | -3,5 | 0,8 | 80,9 | 18,6 | 0,5 | 4,3 |
| Vassoura Preta | -2,5 | -3,3 | 2,2 | 58,1 | 24,3 | 17,6 | 4,4 |  | -4,7 | 0,1 | 0,8 | 42,5 | 51,6 | 5,9 | 3,1 |  | -4,6 | -1,8 | 0,9 | 50,1 | 49,0 | 1,0 | 3,9 |
| BR11-24-156 | -3,7 | -2,3 | 2,1 | 40,4 | 41,4 | 18,3 | 3,9 |  | -3,5 | 1,7 | 0,3 | 73,4 | 19,6 | 7,0 | 3,3 |  | **-4,7** | **-3,1** | **-0,2** | **52,0** | **27,7** | **20,3** | **3,2** |
| Corrente | -4,2 | -2,1 | 2,2 | 34,8 | 46,4 | 18,8 | 3,8 |  | -3,4 | 2,0 | 0,9 | 79,6 | 12,9 | 7,5 | 3,3 |  | -2,9 | -2,6 | 0,3 | 65,5 | 27,3 | 7,2 | 4,9 |
| BRS Formosa | **-3,3** | **-5,1** | **2,5** | **76,3** | **4,1** | **19,6** | **3,1** |  | -4,7 | -0,2 | 0,6 | 40,9 | 58,8 | 0,4 | 3,3 |  | -4,5 | -0,4 | 0,9 | 40,5 | 57,6 | 1,9 | 5,0 |
| BRS Dourada | -3,0 | -1,3 | 3,1 | 47,0 | 50,5 | 2,5 | 5,0 |  | -3,5 | 3,0 | 2,0 | 62,9 | 9,9 | 27,2 | 3,5 |  | -2,8 | -3,9 | 2,1 | 70,7 | 9,0 | 20,2 | 4,8 |
| Cigana Preta | -3,9 | -1,6 | 1,9 | 34,0 | 46,7 | 19,3 | 4,4 |  | -3,0 | 2,1 | 0,0 | 78,0 | 9,6 | 12,4 | 3,7 |  | -2,6 | -2,6 | -0,3 | 61,7 | 24,6 | 13,8 | 5,3 |
| Correntão | -3,6 | -1,0 | 2,5 | 36,8 | 52,0 | 11,2 | 4,9 |  | -2,9 | 2,4 | 1,2 | 83,9 | 2,8 | 13,4 | 3,8 |  | -3,2 | -4,1 | 0,9 | 89,9 | 8,8 | 1,3 | 4,2 |
| BRS Gema de ovo | -2,7 | -1,0 | 1,2 | 37,4 | 40,9 | 21,7 | 5,8 |  | -2,4 | 2,0 | 0,2 | 83,0 | 9,1 | 7,9 | 4,3 |  | -2,5 | -3,0 | 0,1 | 68,5 | 21,0 | 10,5 | 5,1 |
| Eucalipto | -2,5 | -1,0 | 3,2 | 48,3 | 49,8 | 1,9 | 5,6 |  | -2,0 | 2,3 | 1,3 | 83,3 | 2,9 | 13,7 | 4,7 |  | -2,2 | -3,1 | 2,1 | 65,3 | 17,8 | 16,9 | 5,6 |
| BRS Verdinha | -3,0 | -2,7 | -0,6 | 35,3 | 23,9 | 40,8 | 5,7 |  | -2,8 | 1,2 | -1,7 | 51,6 | 17,4 | 31,1 | 4,7 |  | -4,5 | -2,4 | -1,3 | 40,5 | 29,3 | 30,2 | 4,1 |
| IAC-90 | -1,2 | -3,0 | 2,6 | 66,4 | 24,8 | 8,9 | 5,6 |  | -2,1 | -1,1 | 1,3 | 51,5 | 40,5 | 8,0 | 5,9 |  | -2,9 | -0,2 | 0,9 | 50,1 | 48,4 | 1,6 | 6,2 |
| ID | -6,4 | -5,0 | 3,3 |  |  |  |  |  | -6,6 | 2,5 | 0,6 | - | - | - | - |  | -7,4 | -4,5 | 0,8 |  |  |  |  |
